# The *Drosophila* ZNRF1/2 homologue, detour, interacts with HOPS complex and regulates autophagy

**DOI:** 10.1038/s42003-024-05834-1

**Published:** 2024-02-15

**Authors:** Shannon Nicolson, Jantina A. Manning, Yoon Lim, Xin Jiang, Erica Kolze, Sonia Dayan, Ruchi Umargamwala, Tianqi Xu, Jarrod J. Sandow, Andrew I. Webb, Sharad Kumar, Donna Denton

**Affiliations:** 1 Centre for Cancer Biology, University of South Australia, Adelaide, SA 5001 Australia; 2 Faculty of Health and Medical Sciences, The University of Adelaide, Adelaide, SA 5001 Australia; 3 The Walter and Eliza Hall Institute of Medical Research, Parkville, VIC 3052 Australia; 4 Department of Medical Biology, University of Melbourne, Parkville, VIC 3052 Australia

**Keywords:** Macroautophagy, Disease model

## Abstract

Autophagy, the process of elimination of cellular components by lysosomal degradation, is essential for animal development and homeostasis. Using the autophagy-dependent *Drosophila* larval midgut degradation model we identified an autophagy regulator, the RING domain ubiquitin ligase CG14435 (detour). Depletion of *detour* resulted in increased early-stage autophagic vesicles, premature tissue contraction, and overexpression of detour or mammalian homologues, ZNRF1 and ZNRF2, increased autophagic vesicle size. The ablation of *ZNRF1* or *ZNRF2* in mammalian cells increased basal autophagy. We identified detour interacting proteins including HOPS subunits, deep orange (dor/VPS18), Vacuolar protein sorting 16A (VPS16A), and light (lt/VPS41) and found that detour promotes their ubiquitination. The *detour* mutant accumulated autophagy-related proteins in young adults, displayed premature ageing, impaired motor function, and activation of innate immunity. Collectively, our findings suggest a role for detour in autophagy, likely through regulation of HOPS complex, with implications for healthy aging.

## Abstract (web summary)

The *Drosophila* ubiquitin ligase detour and its mammalian homologs ZNRF1/2 are modulators of autophagy that interact with subunits of the HOPS complex and are required for healthy aging.

## Introduction

Macroautophagy (hereafter referred to as autophagy) is a lysosomal degradation pathway that provides cellular quality control by removing cytoplasmic cargos including dysfunctional organelles, protein aggregates and pathogens^1,2^. The material is engulfed by a double membrane vesicle, the autophagosome, that fuses with the lysosome where the contents are degraded. Disruption of autophagy, including the age-dependent decline in activity, contributes to the pathogenesis of several human diseases^2–5^. In particular, genetic studies have revealed that defective autophagy causes progressive neurodegenerative phenotypes highlighting the importance of autophagy for the clearance of abnormal protein aggregates^6–12^. Conversely, enhanced autophagy can result in increased lifespan supporting a role for autophagy in promoting neuronal health^13,14^. This highlights the critical function of autophagy in neuronal homeostasis and healthy aging. Identifying new regulators of this process has the potential to enable modulation of autophagy to protect against disease.

An integral step in autophagic flux is the fusion of the autophagosome with the lysosome, forming autolysosomes, regulated by the homotypic fusion and vacuole protein sorting (HOPS) tethering complex^15,16^. The conserved HOPS complex comprises Vacuolar Protein Sorting 11 (VPS11), VPS16, VPS18, VPS33, that are also common subunits of class C core vacuole/endosome tethering CORVET complex, and VPS39 and VPS41, that are unique to HOPS complex^17,18^. In addition to mediating fusion between autophagosome and lysosome, HOPS complex also regulates fusion of endosomes with lysosomes. The depletion of VPS41 impairs HOPS-dependent delivery of endocytic cargo to lysosomes and causes a defect in autophagic flux^19,20^. A block in autophagic flux is also observed upon knockdown or mutation of other HOPS subunits in mammalian cells^14,16,21,22^. In *Drosophila*, cells depleted of each HOPS subunit have impaired autophagic flux^20^. Recently, mutations in VPS41 were identified in patients displaying neurodegenerative disease, representing a new class of lysosomal disorders^23^. Thus, regulation of the rate of fusion between the autophagosome and lysosome is important in maintaining cellular homeostasis and normal physiology. However, many aspects regarding the regulation of autophagy and the interplay with the endolysosomal network in a physiological setting remain poorly understood.

Autophagy plays context-dependent roles during development and has important functions under both basal conditions and in response to stress. The induction of autophagy in response to stress needs to occur rapidly and is tightly controlled by post-translational modifications (PTMs). A key mechanism for regulation of autophagy is ubiquitination of several core components of the autophagy machinery (e.g., ULK1 and PI3K complexes)^24,25^. Ubiquitin ligases (E3s), together with their interacting proteins, regulate autophagy-related proteins and regulatory components to fine-tune autophagic flux, not only through ubiquitin-mediated proteasomal degradation but also by non-degradative ubiquitin signals. For example, the kinase activity of ULK1, required for autophagy induction, is regulated by the RING-type E3s, tumour necrosis factor receptor (TNFR)–associated factor 6 (TRAF6) and Tripartite motif-containing 32 (TRIM32), which interact with ULK1 via Autophagy And Beclin 1 Regulator 1 (AMBRA1)^26,27^. AMBRA1 is also a substrate adaptor of CUL4 E3s and both AMBRA1 CUL4 ligase and TRAF6 mediate K63-ubiquitination of Beclin 1 (Atg6), promoting autophagy^28,29^. Contrary to TRAF6, the de-ubiquitinating enzyme TNFAIP3/A20 (TNF alpha induced protein 3) reduces K63-linked ubiquitination of Beclin 1, limiting the autophagy response^28^. As with autophagy initiation, E3s plays an important role in autophagy termination. Following autophagy induction, AMBRA1 phosphorylation by ULK1 results in its dissociation from the CUL adaptor DNA damage-binding protein 1 (DDB1) and subsequent stabilisation. AMBRA1 is then free to positively regulate autophagy by increasing ULK1 activity and decreasing mTOR activation^27,30^. Following prolonged starvation, AMBRA1 is ubiquitinated by DDB1/CUL4 and degraded leading to autophagy termination^30^. Autophagy termination is also regulated by Cullin E3 complexes. The K48-linked ubiquitination of ULK1 by CUL3 with adaptor KLHL20, mediates its proteasomal degradation as well as the degradation of other autophagy machinery components^31^.

Although our understanding of the role of ubiquitination in regulation of autophagy has greatly increased, from phagophore nucleation to autophagosome fusion with the lysosome and termination, there are still outstanding questions to be answered. How is the crosstalk between autophagy and other cellular pathways regulated, in particular between the other vesicle pathways, and what roles do E3s have in modulating this? Thus, it is of great importance to identify the E3s involved in regulatory steps of autophagy.

The degradation of the *Drosophila* larval intestine is dependent on autophagy, yet surprisingly only a subset of the canonical autophagy components are essential for this mode of autophagy^32,33^. This suggests that distinct autophagy regulatory proteins and mechanisms may be required. Ubiquitination is important for autophagy and cell size reduction that accompanies midgut degradation^34^. To identify the enzymes of the ubiquitin system required for regulating autophagy we screened a collection of RNAi lines for defects in midgut degradation. Here, we identified *Drosophila* *detour*, that encodes an uncharacterised Really Interesting New Gene (RING) domain-containing protein homologous to mammalian Zinc and RING Finger 1 and 2 (ZNRF1 and ZNRF2)^35,36^. Both ZNRF1 and ZNRF2 have ubiquitin ligase activity and are highly expressed in the nervous system^37^. Previous studies suggest that ZNRF1 and ZNRF2 are involved in growth signalling and protein synthesis^35,38^. However, currently there is no direct evidence linking ZNRF1 or ZNRF2 to autophagy.

Here, we show that *detour* mutants had increased early-stage autophagic vesicles as well as an accumulation of autophagy-related proteins in young *Drosophila* adults. Overexpression of detour and the mammalian homologues, ZNRF1 and ZNRF2 in the *Drosophila* midgut led to increased autophagic vesicle size. We further show that detour interacts with subunits of the HOPS complex, deep orange (dor/VPS18), Vps16A (VPS16), and light (lt/VPS41), and promotes ubiquitination of these components. This function appears to be conserved in mammals with ZNRF2 interacting with VPS18. The HOPS complex activity has been shown to be important in human disease, hence we examined if detour was important for age-related health. The *detour* mutant adults displayed premature aging, impaired motor function, and activation of innate immunity, suggesting that detour is required for healthy aging. Our data reveal a function of detour in autophagy, likely through an interaction with HOPS complex to regulate autophagic vesicle fusion events, which may play a role in longevity.

## Results

### *Drosophila* detour regulates autophagy

During *Drosophila* metamorphosis, the larval intestinal tissue is degraded by an autophagy-dependent pathway^32,39^. The larval intestine cells in the midgut and gastric caeca begin to increase autophagy levels at the onset of degradation (-4 h relative to puparium formation, h RPF), with high levels of autophagy detected by 2 h RPF^32^. Genetic inhibition of autophagy severely delays midgut contraction resulting in incomplete midgut degradation, while inhibition of apoptosis by blocking caspase activity does not affect degradation^32^. Therefore, identifying factors which affect midgut degradation may uncover novel regulators of autophagy. To do this, we screened a collection of RNA*i* knockdown lines targeting ubiquitination machinery components. The screen utilised larval midgut-specific expression of GFP (using Mex-GAL4 driver) to identify genes, that perturbed gastric caeca and midgut degradation when knocked down. This identified *CG14435*, that we have referred to as *detour (detr)*, encoding a RING domain-containing ubiquitin ligase.

**Fig. 1:**
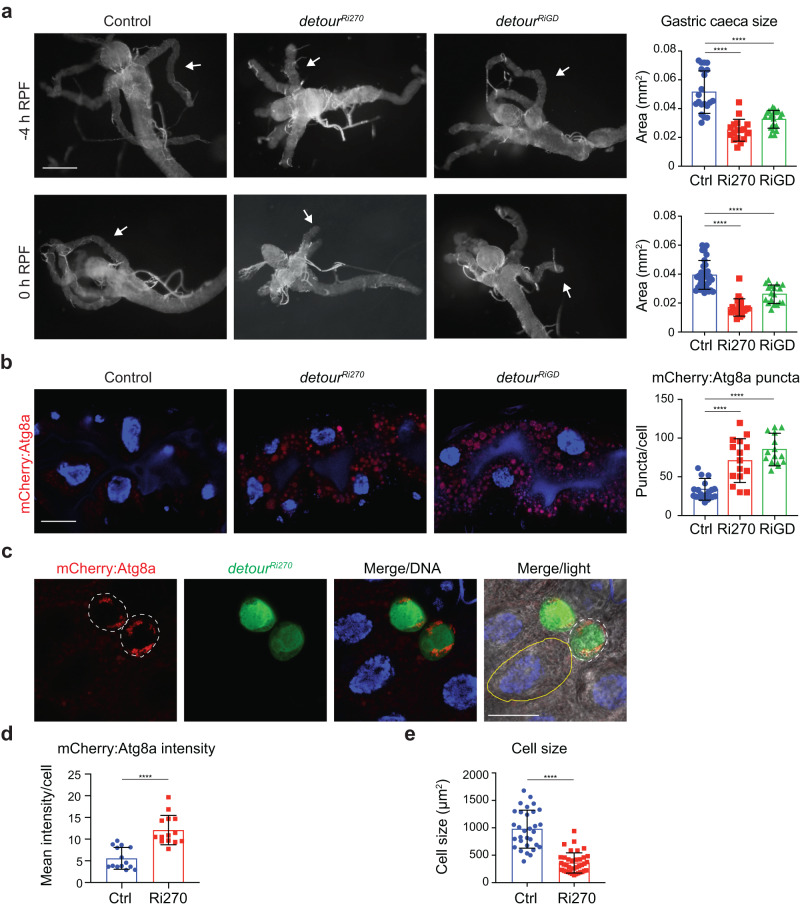
Knockdown of detour increases autophagic vesicles and midgut degradation. **a** Morphology of control (*Mex-GAL4/+; pmCherry-Atg8a/+*), and *detour* knockdown (Ri270 and RiGD) (*Mex-GAL4/+; pmCherry-Atg8a/UAS-detour Ri*) midguts at -4 h and 0 h RPF showed smaller midguts and increased contraction of gastric caeca (arrow). Scale bar = 200 µm. Quantitation of gastric caeca size at -4 h and 0 h RPF. Data presented as area ± SD (****p < 0.0001). **b** The knockdown of *detour* has increased mCherry:Atg8a (red) puncta in the larval midguts at -4 h RPF compared to controls (*Mex-GAL4/+; pmCherry-Atg8a/+*). DNA is stained with Hoechst (blue). Scale bar = 20 μm. Quantitation of puncta at -4 h RPF performed using ImageJ. Data presented as average puncta/cell ± SD (****p < 0.0001). **c** Clone cells of detour knockdown (*hsFLP; pmCherry-Atg8a/+; Act* > *CD2* > *GAL4, UAS-nlsGFP/UAS-detour Ri270*) in the midgut marked by GFP (green) have increased mCherry:Atg8a puncta (red, outlined with dotted line) compared to the neighbouring control cells (non-GFP, outlined with yellow line in merge/light) at -4 h RPF. DNA is stained by Hoechst (blue). Scale bar = 20 μm. **d** Quantitation of mCherry:Atg8a represented as mean intensity/cell ± SD (****p < 0.0001). **e** Quantitation of cell size represented as area ± SD (****p < 0.0001).

To validate the role of *detour* in autophagy-dependent midgut degradation, the original line identified in the screen (*detour*^*Ri270*^) and an additional independent RNAi knockdown line (*detour*^*RiGD*^) were examined. The level of knockdown was verified by qRT-PCR (Supplementary Fig. 1a). The effect of reduced *detour* expression on midgut degradation was assessed by examining the gastric caeca morphology at the early onset (-4 h RPF) and during midgut degradation (0 h RPF) (Fig. 1a). As the larvae cease feeding and begin emptying their gut contents, the midgut condenses slightly and the first distinct morphological change that can be observed is shortening of the gastric caeca. This is followed by a dramatic change in gut morphology between 2 and 4 h RFP, where the gut contracts to a fraction of its original length and the gastric caeca disappear. The degradation of the midgut can be closely followed by the contraction of the gastric caeca^40^. Quantitation of gastric caeca size showed that both *detour*^*Ri270*^ and *detour*^*RiGD*^ had significantly shortened gastric caeca compared to the control at both stages of degradation (Fig. 1a, -4 h RFP and 0 h RPF top and bottom, respectively).

Previous studies have shown a correlation between autophagy and cell size during contraction of the midgut. Inhibition of autophagy results in a failure to undergo cell size reduction, while induction of autophagy decreases cell size^33,34,39^. The effect of reduced *detour* expression on autophagy was examined at the onset of midgut degradation (-4 h RPF) using the pmCherry-Atg8a marker, as previously described^41^. Consistent with the smaller midgut size, *detour*^*Ri270*^ and *detour*^*RiGD*^ knockdown lines showed increased mCherry-Atg8a puncta at -4 h RPF (Fig. 1b). This suggests that reduced levels of detour expression led to increased autophagic vesicles and premature midgut degradation.

To investigate the autophagy defect, we generated mosaic clones in the midgut that give rise to cells knocked down for *detour* (marked by GFP) adjacent to control cells. The *detour*^*Ri270*^ knockdown clone cells displayed increased Atg8a puncta compared to the neighbouring control cells at -4 h RPF (Fig. 1c, d). In addition, the cell size was significantly smaller in *detour* knockdown clones compared to the control (Fig. 1c, e). As the initial genetic screen was based on the larval intestine degradation phenotype, we examined a broader role of detour in autophagy. During larval development, the fat body, an energy storage and utilisation tissue, acts as a sensor of nutrient status. In response to acute starvation, autophagy is induced in the fat body to promote cell survival, while under normal growth conditions, there are very low levels of autophagy^42^. To determine if detour has a broader role in autophagy, its function was examined in the fat body. Clonal knockdown of *detour* in fat body cells resulted in increased autophagic vesicles under normal growth conditions (Supplementary Fig. 1b), a similar phenotype to the midgut. This suggests that detour plays a role in autophagy both in cell death and survival during development.

### *detour* mutations alter autophagy and disrupt developmental tissue degradation

To further examine the role of detour in autophagy, we generated a *detour*^1^ mutant line by imprecise excision of a *Minos* transposon insertion *detour*^*MiET*^ and a revertant line *detour*^*rev*^ by precise excision of the transposable element (Supplementary Fig. 2a). Loss of genomic sequence in *detour*^1^ animals and reversion to wild type open reading frame in *detour*^*rev*^ was confirmed by sequencing the deletion breakpoints and the genomic region, respectively. All lines were backcrossed to *w*^*1118*^ for 6 generations to eliminate any differences in genetic background. The transcript levels were examined by qRT-PCR in adult flies (Supplementary Fig. 2b). The *detour*^1^ is a transcript null allele and the *Minos* transposon insertion line *detour*^*MiET*^ also had significantly reduced mRNA. The precise excision line *detour*^*rev*^ had similar mRNA levels to *w*^*1118*^ wild type (Supplementary Fig. 2b). All lines were homozygous viable and fertile with no externally visible phenotype in adults.

**Fig. 2:**
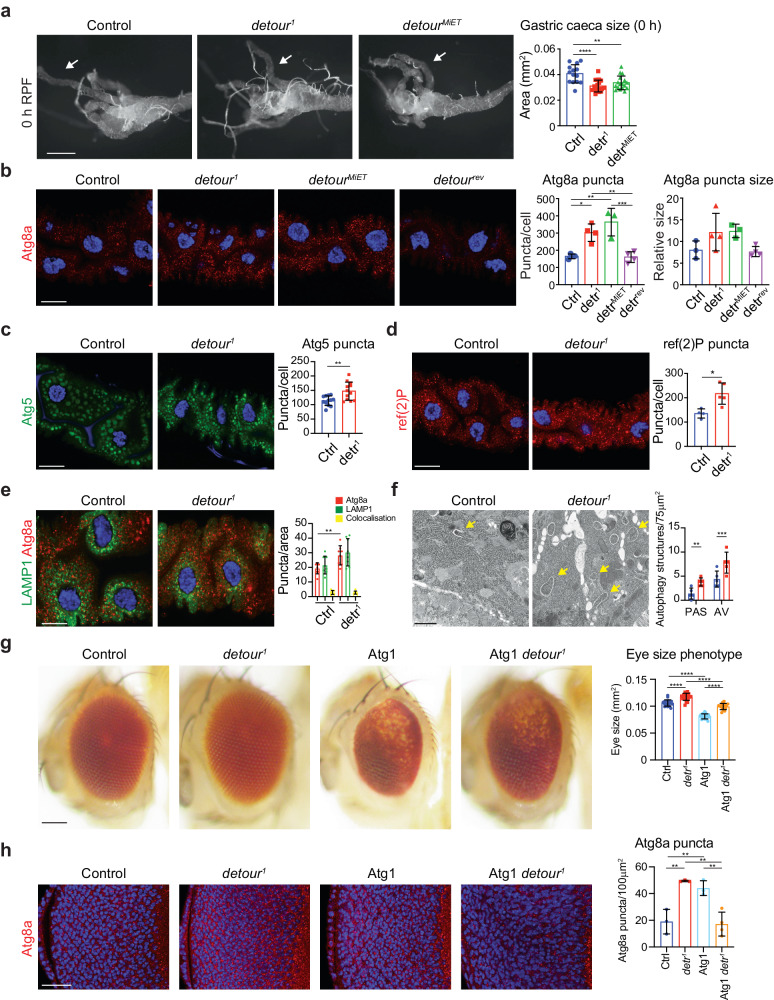
detour mutants have increased autophagic vesicles. **a** Morphology from *detour*^*1*^ and *detour*^*MiET*^ midguts at 0 h RPF shows reduction in gastric caeca size (arrow) compared to control (*w*^*1118*^). Scale bar = 200 µm. Quantitation of the gastric caeca size showing significant reduction in *detour*^*1*^ and *detour*^*MiET*^ compared to control. No significant difference was observed between *detour*^*1*^ and *detour*^*MiET*^. Data presented as area ± SD (****p < 0.0001, **p = 0.0035). **b** The Atg8a immunostaining (red) of midguts at −4 h RPF shows *detour*^*1*^ and *detour*^*MiET*^ have similar levels of Atg8a puncta which are both higher compared to the controls (*w*^*1118*^ and *detour*^*rev*^). DNA is stained by Hoechst (blue). Scale bar = 20 μm. Quantitation of Atg8a positive puncta represented as puncta/cell ± SD (*p < 0.05, **p < 0.01, ***p < 0.001). Quantitation of Atg8a puncta size represented as average relative puncta size. **c** The eGFP:Atg5 (green) puncta in larval midguts at -4 h RPF is increased in *detour*^*1*^ (*detour*^*1*^*/Y; NP1-GAL4/* + *; UAS-eGFP:Atg5/+*) compared to control (*w*^*1118*^*/Y; NP1-GAL4/* + *; UAS-eGFP:Atg5/+*). DNA is stained with Hoechst (blue). Scale bar = 20 μm. Quantitation of puncta represented as average puncta/cell ± SD (**p < 0.01). **d** The ref(2)P immunostaining (red) of midguts at -4 h RPF shows *detour*^*1*^ has increased levels compared to the control (*w*^*1118*^). DNA is stained by Hoechst (blue). Scale bar = 20 μm. Quantitation represented as average puncta/cell ± SD (*p < 0.05). **e** The Atg8a (red) and GFP:LAMP (green, anti-GFP) immunostaining of midguts at −4 h RPF shows *detour*^*1*^ (*detour*^*1*^*/Y; Mex-GAL4, UAS-GFP:LAMP1/+*) has increased Atg8a levels of compared to the control (*w*^*1118*^*/Y; Mex-GAL4, UAS-GFP:LAMP1/* + ). DNA is stained by Hoechst (blue). Scale bar = 10 μm. Quantitation of Atg8a (red), GFP:LAMP (green) and colocalisation (yellow) represented as average puncta/area ± SD (**p < 0.01). **f** Representative TEM images from sections of midgut at -4 h RPF. *detour*^*1*^ midgut cells have increased early autophagic structures (arrows), compared to the control. Scale bar = 1 μm. Quantitation of autophagic structures, pre-autophagosomal structure (PAS) and autophagic vesicle (AV), represented as average structures/area ± SD (**p < 0.01, ***p < 0.001). **g** Over-expression of the Atg1 in the developing eye (*GMR>Atg1*: *GMR-GAL4/* + *; UAS-Atg1/+*) results in a rough eye phenotype, with disruption to patterning, loss of pigmentation and reduced size compared to controls (*GMR w*^*1118*^: *w*^*1118*^*/Y; GMR-GAL4/+* and *GMR detour*^*1*^: *detour*^*1*^*/Y; GMR-GAL4/+*). The Atg1-induced eye phenotype is supressed by *detour*^*1*^ (GMR>Atg1 *detour*^*1*^: *detour*^*1*^*/Y; GMR-GAL4/* + *; UAS-Atg1/+)*, observed by increased red eye pigmentation and eye size. Scale bar = 100 μm. The eye size of *GMR detour*^*1*^ is increased compared to the control (*GMR w*^*1118*^). Quantitation of eye size phenotype represented as eye area ± SD (****p < 0.0001). **h** The Atg8a immunostaining (red) of the eye imaginal disc from wandering third instar larvae shows increased Atg8a in both *GMR detour*^*1*^ and *GMR>Atg1* compared to the control (*GMR w*^*1118*^). The combined *GMR>Atg1 detour*^*1*^ results in reduction of Atg8a puncta compared to both *GMR* *detour*^*1*^ and *GMR>Atg1*. Scale bar = 20 μm. Quantitation of Atg8a positive puncta represented as puncta/area ± SD (**p < 0.01).

Initially, we examined the degradation of the larval midgut to confirm the knockdown phenotypes. Midgut morphology was examined during midgut degradation (0 h RPF) (Fig. 2a). The *detour*^1^ and *detour*^*MiET*^ lines showed significantly reduced gastric caeca size compared to the control (Fig. 2a), consistent with that observed in *detour* knockdown midguts (Fig. 1a). Immunostaining for endogenous Atg8a revealed significantly increased accumulation of Atg8a positive puncta in *detour*^1^ and *detour*^*MiET*^ compared to the controls (Fig. 2b). There was a trend for increased puncta size, however this was not significantly different to the controls (Fig. 2b). We also made use of a deficiency (*Df*) that removes *detour* (and several other genes) to generate transheterozygotes and examined autophagy in -4 h RPF larval midguts. Consistent with the homozygous mutants, immunostaining for endogenous Atg8a revealed a significant increase in Atg8a positive puncta in *detour*^*1*^/*Df* and *detour*^*MiET*^/*Df* compared to the controls (Supplementary Fig. 2c). Hence the *detour* mutants and knockdown lines have similar phenotypes, resulting in increased autophagic vesicles and midgut degradation.

To further assess the increase in autophagic vesicle, we examined Atg5 puncta formation, an early-stage autophagic vesicle marker. While Atg8 remains associated with autophagosomes, Atg5 is associated with phagophores but not with autophagosomes^43^. This showed that there was also an increase in Atg5 positive puncta in the *detour*^*1*^ mutant cells compared to the control (Fig. 2c). In addition, the *Drosophila* p62 autophagic receptor and substrate, refractory to sigma P (ref(2)P), accumulated in *detour* mutant cells compared to control (Fig. 2d). Under normal conditions, ref(2)P is internalised within the autophagosome and degraded by the autolysosome. This suggests that lysosomal degradation of autophagic cargoes may be impaired. This can result from accumulation of autophagosomes or reduced lysosomal function. The function of the lysosome is dependent on an acidic pH for the degradative activity and can be assessed using LysoTracker, a dye that labels acidic vesicles, as well as the processing of lysosomal enzymes, including Cathepsin L. There was no difference in LysoTracker staining in the *detour* mutant compared to control (Supplementary Fig. 2d). In addition, both pro and mature forms of Cathepsin L were detected in the control and *detour* mutant (Supplementary Fig. 2e). This suggests that lysosome function is not altered in *detour* mutant. To determine the rate of autolysosome formation, we examined the colocalisation of Atg8a with GFP-tagged LAMP1 (human LAMP1 fused to GFP detected by immunostaining for GFP). This enables the detection of autophagosomes as Atg8a positive puncta and autolysosomes as dual-labelled Atg8a and LAMP1 positive vesicles^44^ (Fig. 2e). The *detour* mutant cells had an increased number of Atg8a positive puncta, while the number of colocalised Atg8a and LAMP1 positive autolysosomes did not show a corresponding increase (Fig. 2e).

Importantly, ultrastructural analysis showed that the *detour* mutant midgut cells (at -4 h RPF) had an increase in the formation of autophagic vesicles compared to the control based on transmission electron microscope (TEM) analysis (Fig. 2f). The morphology of these vesicles suggests that they may be at a very early stage of autophagic vesicle formation and accumulate in the *detour* mutant intestine cells without an increase in electron-dense lysosomes. The increase in early-stage autophagosomes (isolation membranes) suggests that closure/maturation may be delayed and/or there is an increase in autophagy induction. These data suggest an essential role for detour in biogenesis of autophagic vesicles in intestine cells.

To further examine the role of *detour* in the autophagy pathway we examined whether *detour* genetically interacts with *Atg1*, which is essential for initiating autophagosome formation (during nutrient stress and development). Overexpression of *Drosophila* Atg1 in the developing eye (using GMR-Gal4) induces a high level of autophagy and results in a roughening of the adult eye structure, with disrupted patterning, loss of pigmentation and reduced size compared to the control^45^ (Fig. 2g). We found that the combined *detour*^1^ with Atg1 resulted in a less severe eye phenotype compared to Atg1 alone, with an increase in eye pigmentation and reduced disorganisation of the eye pattern resulting in a larger eye size (Fig. 2g). To examine the cellular basis for this, Atg8a positive vesicles were examined in third instar larval eye imaginal disc that gives rise to the adult eye. This revealed that both *detour*^1^ and Atg1 had increased Atg8a. Consistent with the reduced severity of the eye phenotype, the combined *detour*^1^ Atg1 had Atg8a puncta levels similar to the control (Fig. 2h). This reduction in Atg8a suggest that Atg1 overexpression results in increased Atg8a due to increased induction (increased flux) and *detour* mutant increases Atg8a due to decreased flux with the combination resulting in normal flux. This is also consistent with the role of Atg1 downstream of induction to promote autophagic clearance^46,47^. Interestingly, the adult eye of *detour*^1^ (with GMR alone) displayed an increase in size (Fig. 2g), and this was also observed in *detour*^1^ hemizygous males compared to *w*^*1118*^ males (Supplementary Fig. 2f). The genetic interactions identified between *Drosophila* Atg1 and *detour* supports the role of detour in autophagy. Taken together, this suggests that detour may function to regulate autophagy and that the increased phagophores and autophagosomes in *detour* mutants is due to a block in the late-stage autophagy process and the accumulation of autophagic vesicles.

### Expression of detour and mammalian ZNRF1 and ZNRF2 affects autophagic vesicle size in *Drosophila*

**Fig. 3:**
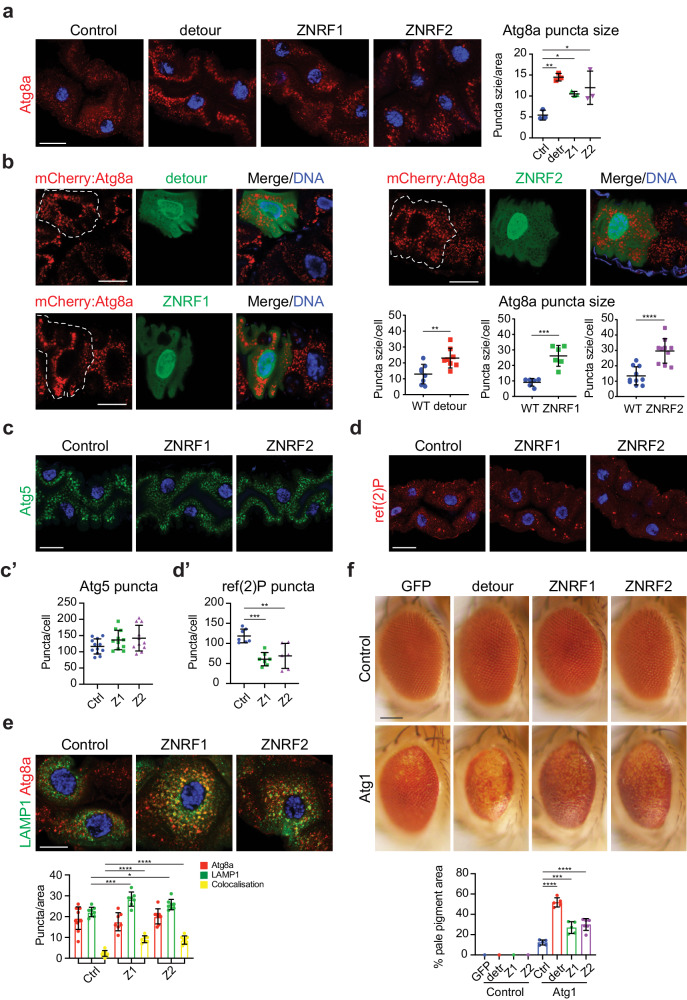
Overexpression of detour, ZNRF1 or ZNRF2 increases autophagic vesicle size. **a** Immunostaining of larval midguts at -4 h RPF overexpressing detour (*Mex-GAL4/* + *; UAS-detour:GFP/* + ), ZNRF1 (*Mex-GAL4/UAS-ZNRF1*) or ZNRF2 (*Mex-GAL4/UAS-ZNRF2*) show increased Atg8a (red) puncta size compared to control (*Mex-GAL4/* + ). DNA is stained with Hoechst (blue). Scale bar = 20 μm. Quantification of Atg8a puncta size, measured in ImageJ. Data presented as average puncta ± SD (*p < 0.05, **p < 0.001). **b** Clone cells overexpressing detour (*hsFLP; pmCherry-Atg8a/+; Act* > *CD2* > *GAL4, UAS-nlsGFP/UAS-detour:GFP*), ZNRF1 (*hsFLP; pmCherry-Atg8a/UAS-ZNRF1; Act* > *CD2* > *GAL4, UAS-nlsGFP/+*) or ZNRF2 (*hsFLP; pmCherry-Atg8a/ UAS-ZNRF2; Act* > *CD2* > *GAL4, UAS-nlsGFP/+*) in the midgut marked by GFP (green) have increased mCherry:Atg8a puncta size (red, clone outlined) compared to the neighbouring control cells at -4 h RPF. DNA is stained by Hoechst (blue). Scale bar = 10 μm. Quantification of mCherry:Atg8a puncta size in GFP-clone cells and neighbouring wildtype cells. Minimum of *n* = 6 cells. Average puncta size/cell ± SD (**p = 0.0059, ***p = 0.0002, ****p < 0.0001). **c** The eGFP:Atg5 (green) puncta in larval midguts at -4 h RPF in *ZNRF1* (*NP1-GAL4/UAS-ZNRF1; UAS-eGFP:Atg5/+*) and ZNRF2 (*NP1-GAL4/UAS-ZNRF2; UAS-eGFP:Atg5/+*) is similar to the control (*NP1-GAL4/* + *; UAS-eGFP:Atg5/+*). DNA is stained with Hoechst (blue). Scale bar = 20 μm. **d** The ref(2)P immunostaining (red) of *ZNRF1* (*Mex-GAL4/UAS-ZNRF1)* and ZNRF2 (*Mex-GAL4/UAS-ZNRF2*) midguts at -4 h RPF shows reduced levels compared to the control (*Mex-GAL4/* + ). DNA is stained by Hoechst (blue). Scale bar = 20 μm. **c’** Quantitation of GFP:Atg5 puncta from (c) represented as average puncta/cell ± SD. **d’** Quantitation of ref(2)P puncta from **d** represented as average puncta/cell ± SD (**p < 0.01, ***p < 0.001). **e** The Atg8a (red) and GFP:LAMP (green, anti-GFP) immunostaining of midguts at −4 h RPF shows ZNRF1 (*Mex-GAL4, UAS-GFP:LAMP1/UAS-ZNRF1*) and ZNRF2 (*Mex-GAL4, UAS-GFP:LAMP1/UAS-ZNRF2*) have increased colocalisation (yellow) compared to the control (*Mex-GAL4, UAS-GFP:LAMP1/* + ). DNA is stained by Hoechst (blue). Scale bar = 10 μm. Quantitation of Atg8a (red), GFP:LAMP (green) and colocalisation (yellow) represented as average puncta/area ± SD (**p < 0.01). **f** Over-expression of detour (*GMR-GAL4/* + *; UAS-Atg1/UAS-detour:GFP)*, ZNRF1 (*GMR-GAL4/UAS-ZNRF1; UAS-Atg1/+)* or ZNRF2 (*GMR-GAL4/UAS-ZNRF2; UAS-Atg1/+)* in the developing eye results in mild patterning disruption compared to control (*GMR-GAL4/UAS-eGFP)* (top panels). Over-expression of the Atg1 in the developing eye (*GMR-GAL4/* + *; UAS-Atg1/+*) results in a rough eye phenotype, with disruption to patterning, loss of pigmentation and reduced size compared to control. The Atg1-induced eye phenotype is enhanced by overexpression of detour (*GMR-GAL4/* + *; UAS-Atg1/UAS-detour:GFP)*, ZNRF1 (*GMR-GAL4/UAS-ZNRF1; UAS-Atg1/+)* or ZNRF2 (*GMR-GAL4/UAS-ZNRF2; UAS-Atg1/+)*, observed by the loss of red eye pigmentation (bottom panels). Scale bar = 100 μm. Quantitation of the pale eye pigmentation phenotype represented as percentage ± SD (****p < 0.0001).

The mammalian homologues of *Drosophila* detour are ZNRF1 and ZNRF2, closely related members of the RING superfamily of ubiquitin ligases. Previous studies suggest that ZNRF1 and ZNRF2 are involved in growth signalling, and ubiquitination^35,38,48,49^. However, there is no direct evidence for a specific role of ZNRF1 or ZNRF2 in autophagy. To examine the potential conservation of autophagy regulatory function, we overexpressed detour, ZNRF1 or ZNRF2 in the *Drosophila* larval midgut. Although this did not affect the contraction of the gastric caeca (Supplementary Fig. 3a), it resulted in an increase in Atg8a positive puncta size, both immunostaining and live mCherry-Atg8a, at -4 h RPF (Fig. 3a; Supplementary Fig. 3b).

To investigate the increased autophagic vesicle size, we generated clones in the midgut that give rise to cells that overexpressed detour, ZNRF1 or ZNRF2 (marked by GFP) adjacent to control cells (Fig. 3b). The detour expressing cells were similar in size compared to the neighbouring wild type cells but had increased size of mCherry-Atg8a puncta at -4 h RPF (Fig. 3b). Likewise, clone cells expressing ZNRF1 or ZNRF2 in the midgut were similar in size compared to the neighbouring control cells and also displayed increased size of mCherry-Atg8a puncta at -4 h RPF (Fig. 3b). These results suggest that overexpression of detour, ZNRF1 or ZNRF2 causes increased autophagic vesicle size. This was surprising, as the ablation of *detour* results in increased autophagic vesicles, with early stage phagophores and autophagosomes, while the overexpression promotes accumulation of larger autophagic vesicles. This suggests that increasing or decreasing detour activity alters autophagic flux.

To further assess the increase in Atg8a positive puncta, the level of GFP:Atg5 positive puncta was examined in ZNRF1 and ZNRF2 expressing midgut cells (not in detour:GFP due GFP-tag). There was no increase in Atg5 positive puncta in the ZNRF1 or ZNRF2 overexpression cells compared to the control (Fig. 3c, c’). In addition, ref(2)P levels were reduced in ZNRF1 or ZNRF2 expressing cells compared to control (Fig. 3d, d’), suggesting an increase in degradation by the autolysosome. To determine the rate of autolysosome formation, we examined the colocalisation of Atg8a with GFP:LAMP1. The ZNRF1 and ZNRF2 expressing cells had an increased number of colocalised Atg8a and LAMP1 positive autolysosomes compared to the control (Fig. 3e). In addition, the total number of lysosomes was also increased, supporting an increase in autolysosomes.

Given the genetic interaction between *detour* and Atg1, we further examined the role of detour, ZNRF1 and ZNRF2 in autophagy by examining the genetic interaction with Atg1 (Fig. 3). We found that the co-expression of detour, ZNRF1 or ZNRF2 with Atg1 resulted in a more severe eye phenotype, compared to Atg1 alone, with a further decrease in eye pigmentation and disorganisation of the eye pattern (Fig. 3f). The expression of detour, ZNRF1 or ZNRF2 alone in the eye displayed only a very minor disruption to patterning with no pigment loss (Fig. 3f). The genetic interactions identified between *Drosophila* Atg1 and ZNRF1, ZNRF2 and detour supports their conserved role in the regulation of autophagy, and suggests that they act to further promote the activity of the autophagy pathway.

### ZNRF1 and ZNRF2 maintain basal autophagy

To investigate the role of ZNRF1 and ZNRF2 in autophagy in mammals, CRISPR-Cas9 knockout (KO) HeLa cells were generated. DNA sequencing analysis confirmed biallelic mutations, 1 bp insertion and 56 bp deletion for *ZNRF1* KO clone (B8) (Supplementary Fig. 4a–c). The introduction of these insertion-deletions caused a frameshift mutation in *ZNRF1* (Supplementary Fig. 4b, c), which resulted in reduced mRNA expression (Supplementary Fig. 4d). Immunoblot analysis and immunostaining confirmed loss of ZNRF2 protein in *ZNRF2* KO clone (D9) (Supplementary Fig. 4e, f).

**Fig. 4:**
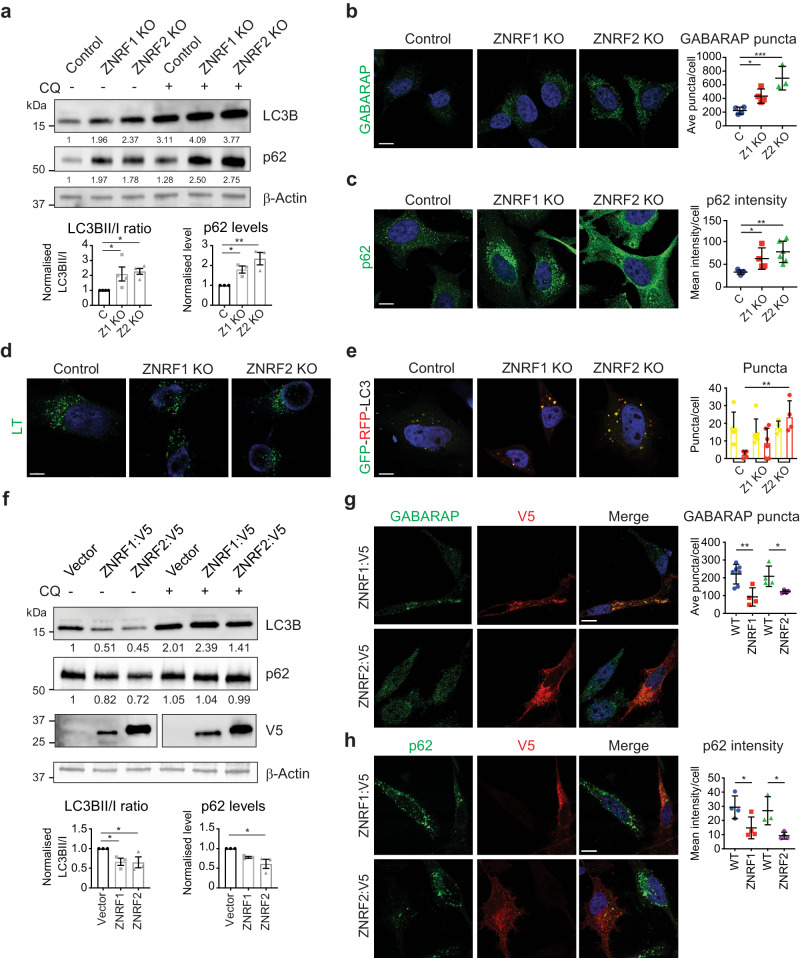
ZNRF1 and ZNRF2 maintain basal autophagy in HeLa cells. **a** Immunoblot analysis of LC3B and p62 protein levels in whole cell lysates from control, *ZNRF1* KO and *ZNRF2* KO HeLa cells with or without 4 h of chloroquine (CQ) treatment. β-Actin used as a loading control. Numbers represent quantitation of the shown immunoblot normalised to β-Actin and compared to Control. Three immunoblot replicates were quantified, normalised to β-Actin and graphed as relative expression to WT cell line. Average ± SD (*p < 0.05, **p < 0.01). **b** Immunostaining of control, *ZNRF1* and *ZNRF2* KO HeLa cells with GABARAP antibody (green) merged with nuclei stained by Hoechst (blue). Scale bar = 10 µm. Quantification of GABARAP-positive puncta number using ImageJ software ± SD (*p < 0.05, **p = 0.001). **c** Immunostaining of control, *ZNRF1* and *ZNRF2* KO HeLa cells with p62 antibody (green) merged with nuclei stained by Hoechst (blue). Scale bar = 10 µm. Quantification of p62 fluorescence intensity using photoshop histogram function ± SD (**p < 0.01). **d** LysoTracker staining of control, *ZNRF1* and *ZNRF2* KO HeLa cells merged with nuclei stained by Hoechst (blue). Scale bar = 10 µm. **e** Control, *ZNRF1* and *ZNRF2* KO HeLa cells transfected with GFP-RFP-LC3 merged with nuclei stained by Hoechst (blue). Scale bar = 10 µm. Quantification of GFP-RFP-LC3 (yellow) and RFP-LC3 (red) fluorescence represented as puncta/cell ± SD (**p < 0.01). **f** Immunoblot analysis of LC3B and p62 protein levels in whole cell lysates transfected with control (vector), ZNRF1:V5 and ZNRF2:V5 HeLa cells with or without 4 h of chloroquine (CQ) treatment. β-Actin used as a loading control. Numbers represent quantitation of the shown immunoblot normalised to β-Actin and compared to Control. Three immunoblot replicates were quantified, normalised to β-Actin and graphed as relative expression to WT cell line. Average ± SD (*p < 0.05, **p < 0.01). **g** Immunostaining of HeLa cells transfected with ZNRF1:V5 and ZNRF2:V5 with GABARAP antibody (green), V5 (red), and merged with nuclei stained by Hoechst (blue). Scale bar = 10 µm. Quantification of GABARAP-positive puncta number using ImageJ software ± SD (*p < 0.05, **p = 0.001). **h** Immunostaining of HeLa cells transfected with ZNRF1:V5 and ZNRF2:V5 with p62 antibody (green), V5 (red), and merged with nuclei stained by Hoechst (blue). Scale bar = 10 µm. Quantification of p62 fluorescence intensity using photoshop histogram function ± SD (**p < 0.01).

The basal level of autophagy was examined in the *ZNRF1* and *ZNRF2* KO HeLa cell lines. Under normal growth conditions, *ZNRF1* and *ZNRF2* KO cells had increased LC3B lipidation (LC3-II) compared to control cells (Fig. 4a). The autophagy adaptor p62 (*Drosophila* ref(2)P) recruits ubiquitinated cargoes to autophagic membranes. Importantly, ZNRF1 and ZNRF2 KO cells had significantly increased p62 level compared to control cells (Fig. 4a). Moreover, the presence of chloroquine to block lysosomal function further increased LC3B and p62 levels (Fig. 4a).

Consistent with this, there was an increase in autophagic vesicles (GABARAP) in the *ZNRF1* and *ZNRF2* KO cells (Fig. 4b). Similarly, there was increased p62 in both the KO cell lines, with a significantly increased fluorescence intensity in the *ZNRF1* and *ZNRF2* KO cells compared to the control cells (Fig. 4c). In the presence of chloroquine, p62 immunostaining was increased further in the ZNRF1 and ZNRF2 KO cells (Supplementary Fig. 4g). This suggests that there is an increase in basal autophagy in the ZNRF1 and ZNRF2 KO cells. Increased p62 and lipidated LC3B levels can also result from reduced lysosomal function. There was no alteration to LysoTracker staining in the ZNRF1 and ZNRF2 KO cells compared to the control (Fig. 4d). Similarly, there was similar levels of the lysosomal enzyme Cathepsin L activity between control, ZNRF1 KO and ZNRF2 KO cells (Supplementary Fig. 4h) suggesting that lysosome function was not affected. To determine the rate of autolysosome formation, a tandem-tagged GFP-RFP-LC3 reporter was used where dual fluorescence of both GFP and RFP reflects autophagosomes and RFP alone reflects autolysosomes. In ZNRF1 and ZNRF2 KO cells transfected with the reporter, there was an increase in the total number of autophagic vesicles compared to the control (Fig. 4e). There was a significant increase in autolysosomes (RFP alone) in the ZNRF2 KO cells compared to the controls (Fig. 4e). These data show that there is an increase in autophagic flux in *ZNRF1* and *ZNRF2* KO HeLa cells.

We next determined if the expression of ZNRF1 and ZNRF2 was sufficient to reduce the levels of autophagy. The basal level of autophagy was examined in ZNRF1 and ZNRF2 over-expression in HeLa cells. Under normal growth conditions, expression of ZNRF1 and ZNRF2 resulted in reduced LC3B compared to the control cells (Fig. 4f). The level of p62 was significantly reduced in ZNRF2 cells compared to control cells (Fig. 4f). In the presence of chloroquine there was an accumulation of LC3B and p62 (Fig. 4f). The overexpression of ZNRF1 and ZNRF2 reduced the levels of autophagic vesicles (GABARAP) (Fig. 4g). Similarly, there was a decrease in the abundance of p62 in both in the ZNRF1 and ZNRF2 expressing cells (Fig. 4h). These findings are consistent with our observations in *detour* mutant flies and suggest that there is altered autophagic flux in the absence of ZNRF1 and ZNRF2. Similar to that observed in *Drosophila*, ZNRF1 and ZNRF2 may also regulate the rate of autophagosome biogenesis in higher organisms.

### Identification of detour interacting proteins

**Fig. 5:**
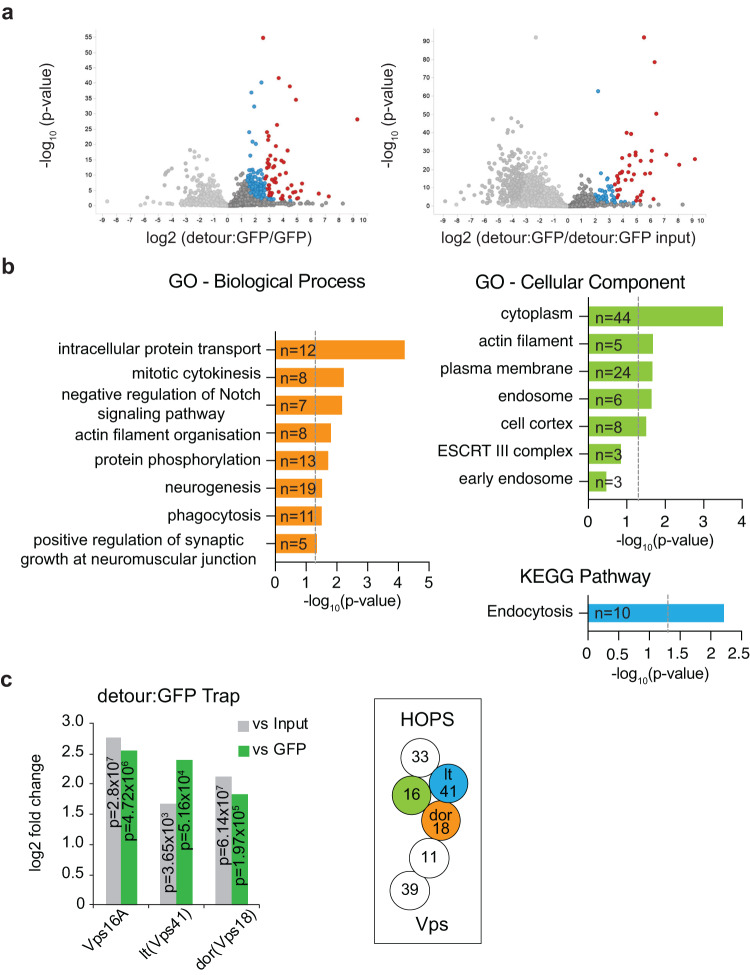
Identification of detour interacting proteins. **a** Quantitative analysis of detour proteins interacting. Volcano plots showing the estimated log2 fold changes versus the -log10 *p*-values for each protein identified as interacting with detour:GFP compared to GFP or detour:GFP input (before immunoprecipitation). Proteins that are highly (4-fold change) and moderately enriched (2-fold change) are shown in red and blue respectively. *p*-values determined using Mann–Whitney *U*-test and Benjamini–Hochberg correction. **b** Functional enrichment analysis of candidate detour-interacting proteins. Gene Ontology (GO) analysis of biological processes and cellular components associated with detour:GFP-interacting proteins (*n* = 157). Pathway enrichment analysis (Kyoto Encyclopaedia of Genes and Genomes, KEGG) of detour:GFP-interacting proteins (*n* = 157). Vertical dotted line indicates significance cut-off (-log10 of the Benjamini–Hochberg corrected *p*-value of 0.05), *p*-values Fisher’s exact test. **c** Identification of Vps16A, lt/VPS41 and dor/VPS18 from detour:GFP IP-MS. Schematic representation of HOPS complex.

Loss of *detour, ZNRF1* or *ZNRF2* resulted in altered autophagic flux (Figs. 1, 2, 4), yet the mechanisms by which detour functions in autophagy is not established. To provide insights into its role in autophagy, we identified proteins that interact with detour using immunoprecipitation and mass spectrometry (IP–MS) of soluble protein extracts from *Drosophila* S2 cells that expressed detour:GFP or GFP alone. Pairwise comparisons between detour:GFP and GFP, and between detour:GFP and the detour:GFP input identified 157 candidate interacting proteins. These proteins were enriched by more than four-fold compared to GFP, had a *p*-value of ≤0.05 and were identified in at least two of the three replicates (Fig. 5a).

Bioinformatic analysis of the data revealed biological processes (BP) associated with detour that may be relevant to its role in autophagy (Fig. 5b). The putative detour-interacting proteins were subjected to gene ontology (GO)-term enrichment analysis. Of these proteins, 97 were assigned to a BP annotation by DAVID. These proteins were associated with eight BP annotations with intracellular protein transport (GO:0006886) being the most enriched. The other BP annotations included mitotic cytokinesis (GO:0000281), negative regulation of Notch signalling pathway (GO:0045746), actin filament organisation (GO:0007015), protein phosphorylation (GO:0006468), neurogenesis (GO:0022008), phagocytosis (GO:0006909) and positive regulation of synaptic growth at the neuromuscular junction (GO:0045887).

The cellular component analysis identified that the putative detour-interacting proteins were significantly associated with the cytoplasm, actin filament and membranous organelles including the endosome (GO:0005768) (Fig. 5b). Consistently, pathway enrichment analysis using the KEGG database identified endocytosis (Fig. 5b). Intriguingly, three HOPS complex subunits, Vacuolar protein sorting 16A (Vps16A), deep orange (dor) and light (lt), were identified (Fig. 5c). These proteins are the *Drosophila* homologues of the HOPS complex components VPS16, VPS18 and VPS41 respectively. While Vps16A and dor are common subunits of the HOPS and CORVET complexes, lt is a HOPS-specific component^50^.

### detour interacts with HOPS complex subunits dor, Vps16A and lt

**Fig. 6:**
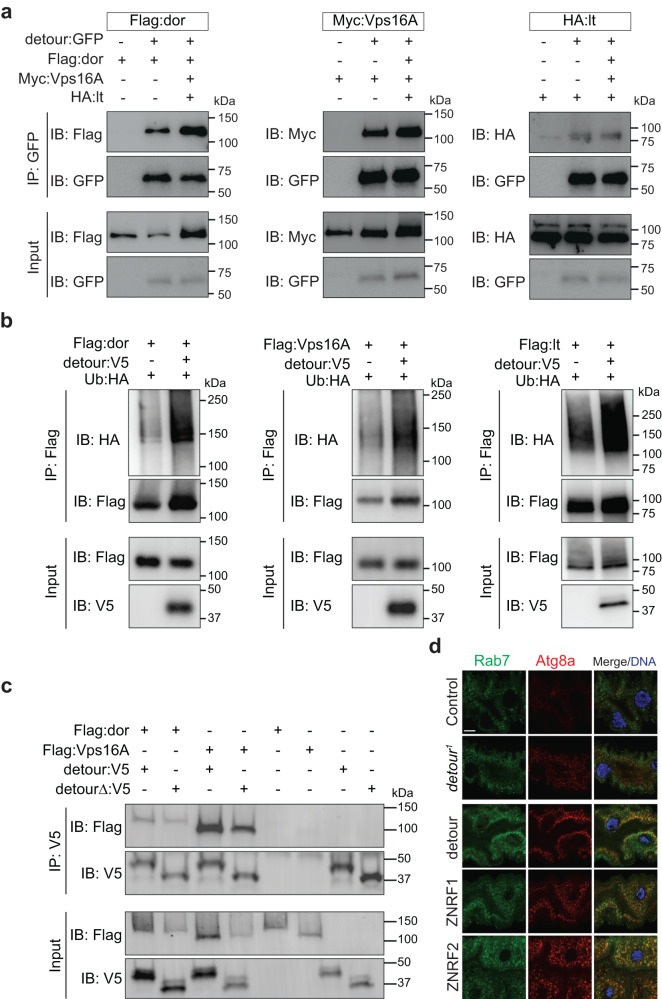
detour interacts with the *Drosophila* HOPS complex. **a** SL2 cells were co-transfected with GFP-tagged detour and Flag-tagged dor, Myc-tagged Vps16A and HA-tagged lt as indicated and subjected to immunoprecipitation (IP) with anti-GFP antibody. Proteins were separated by SDS-PAGE and immunoblotted (IB) with anti-GFP antibody and anti-Flag, anti-Myc or anti-HA antibody. Input controls were 5% of each protein lysate. **b** In the presence of detour there is increased ubiquitination of dor, Vps16A and lt in SL2 cells co-transfected with detour, HA-tagged Ub and Flag-tagged dor or Flag-tagged Vps16A or Flag-tagged lt as indicated. IPs were carried out using anti-Flag and IB with anti-Flag, anti-HA and anti-V5 antibody. **c** Lysates from SL2 cells co-transfected with V5-tagged detour (detour:V5) or V5-tagged detour RING deletion (detourΔ:V5) and Flag-tagged dor or Flag-tagged Vps16A were subjected to immunoprecipitation (IP) with anti-V5 antibody. Proteins were separated by SDS-PAGE and immunoblotted (IB) with anti-Flag and anti-V5 antibody. Input controls were 5% of each protein lysate. **d** Atg8a and Rab7 staining shows increased Atg8a puncta and enlarged Rab7 positive vesicles in *detour* mutants. Overexpression of detour, ZNRF1 or ZNRF2 have enlarged Atg8a puncta surrounded by Rab7. The Rab7 in detour:GFP was detected with 647 and is coloured green. Scale bar = 20 μm.

We further examined the interaction between detour and dor, Vps16A and lt by co-immunoprecipitation (co-IP) (Fig. 6a; Supplementary Fig. 5a). To verify the interaction between detour and dor, we performed a co-IP from *Drosophila* S2 cell lysates co-expressing detour:GFP and Flag:dor. Flag:dor was detected when detour was pulled down (Fig. 6a; Supplementary Fig. 5a). We also confirmed an interaction between detour and Vps16A in cells by co-IP using S2 cells co-expressing detour:GFP and Myc:Vps16A (Fig. 6a; Supplementary Fig. 5a). Similarly, detour and lt interaction was verified by co-IP in S2 cells co-expressing detour:GFP and HA:lt (Fig. 6a; Supplementary Fig. 5a). None of the HOPS subunits co-IPed with GFP alone (Supplementary Fig. 5a). When all subunits were expressed together, IP of detour was able to pull down dor, Vps16A and lt, indicating an interaction of detour with the HOPS complex (Fig. 6a). Finally, we examined ubiquitination of HOPS components using full-length detour and dor, Vps16A and lt, demonstrating increased ubiquitination of dor, Vps16A and lt (Fig. 6b). The C-terminal region of detour contains a RING domain, the putative E3 domain (Supplementary Fig. 2a). To determine the role of the RING in the interaction with the HOPS components we generated a detour C-terminal deletion (aa 257-303 deleted). Interestingly, the RING domain deletion of detour was still able to interact with dor and Vps16A, albeit at reduced levels (Fig. 6c). While there was decreased dor and Vps16A in the presence of detour RING domain deletion, there also appeared to be reduced ubiquitination of dor and Vps16A compared to full-length detour (Supplementary Fig. 5b). It is also important to consider that dor contains a RING domain that in VPS18 has been shown to function of an E3^51^. Our data supports a role for detour in the regulation of the HOPS complex ubiquitination, either directly or indirectly by regulation of another ubiquitin ligase.

The HOPS membrane tethering complex is not only required for the fusion between late endosomes and lysosomes, but it also participates in autophagy by mediating the fusion between autophagosomes and lysosomes^16,17^. Having identified a role for detour in autophagy and an interaction between detour and HOPS complex, we examined double labelling of Atg8a autophagy marker with Rab7 to mark membrane-bound compartments of the late endosomes and lysosomes. In the *detour* mutant midguts, Rab7 had minimal colocalisation with the small Atg8a positive puncta in -4 h RPF midguts (Fig. 6d). This was in stark contrast to the overexpression of detour, ZNRF1 or ZNRF2 in the *Drosophila* larval midgut that resulted in an increased accumulation of large Rab7 positive vesicles that enclosed large Atg8a positive puncta at -4 h RPF (Fig. 6d). This suggests that detour activity promotes HOPS complex function in the fusion of autophagic vesicles with lysosomes and/or late endosomes.

### *Drosophila* deep orange and light are essential for autophagy in the larval midgut

**Fig. 7:**
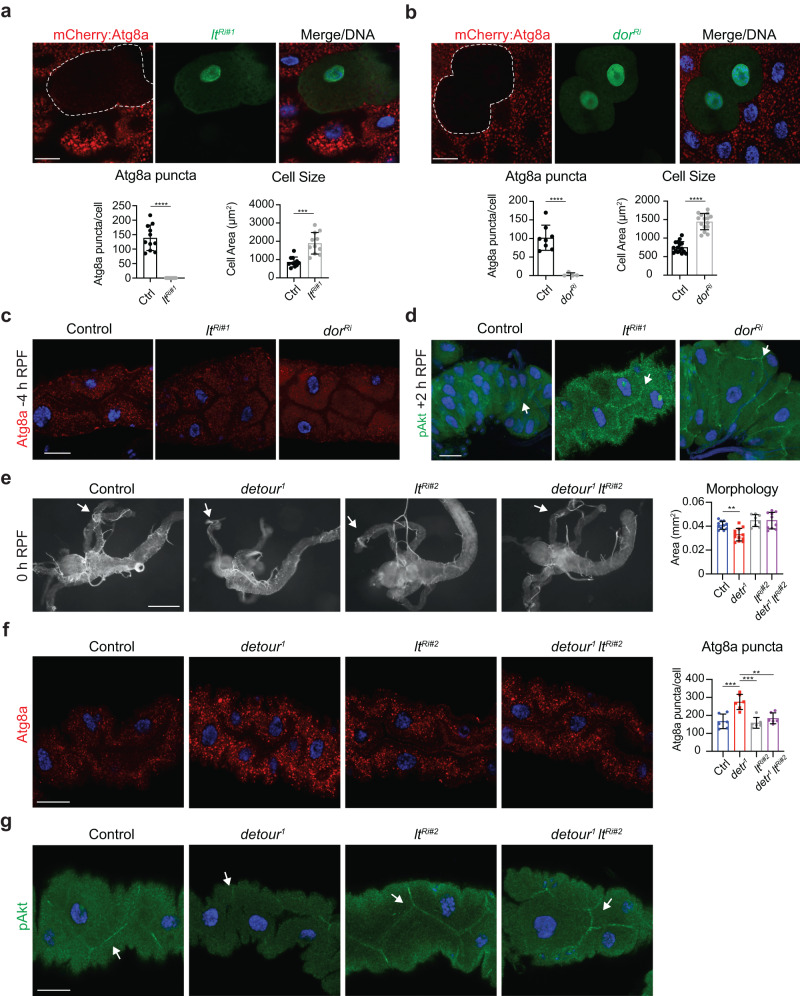
*detour* genetically interacts with *light*. **a** Clone cells knockdown for *lt* (*hsFLP; pmCherry-Atg8a/UAS-ltRi*#1*; Act* > *CD2* > *GAL4, UAS-nlsGFP/+)* in the midgut marked by GFP (green) has decreased mCherry:Atg8a puncta size (red, clone outlined) and increased cell size compared to the neighbouring control cells at -4 h RPF. DNA is stained by Hoechst (blue). Scale bar = 10 μm. Quantification of mCherry:Atg8a puncta size represented as average puncta size/cell ± SD. Quantitation of cell size represented as area ± SD (****p < 0.0001). **b** Clone cells knockdown for *dor* (*hsFLP; pmCherry-Atg8a/+; Act* > *CD2* > *GAL4, UAS-nlsGFP/UAS-dorRi*) in the midgut marked by GFP (green) has decreased mCherry:Atg8a puncta size (red, clone outlined) and increased cell size compared to the neighbouring control cells at -4 h RPF. DNA is stained by Hoechst (blue). Scale bar = 10 μm. Quantification of mCherry:Atg8a puncta size represented as average puncta size/cell ± SD. Quantitation of cell size represented as area ± SD. (***p < 0.001, ****p < 0.0001). **c** The Atg8a immunostaining (red) of midguts at −4 h RPF from knockdown of *lt* (*Mex-GAL4/UAS-ltRi*#1, strong knockdown line) and *dor* (*Mex-GAL4/* + *; UAS-dorRi/+*) compared to controls (*Mex-GAL4/* + ). DNA is stained with Hoechst (blue). Scale bar = 20 μm. **d** The phosphorylated Akt (pAkt) immunostaining (green) of midguts at +2 h RPF shows cortical localisation (arrow) in *lt* (*Mex-GAL4/UAS-ltRi*#1*; pmCherry-Atg8a/+*) and *dor* knockdown (*Mex-GAL4/+; pmCherry-Atg8a/UAS-dorRi*) which is reduced in the control (*Mex-GAL4/+; pmCherry-Atg8a/+*). DNA is stained by Hoechst (blue). Scale bar = 20 μm. **e** Morphology of control (*w*^*1118*^), *detour*^*1*^, the weak *lt*^*Ri#2*^ knockdown (*Mex-GAL4/* + *; UAS-ltRi#2*/+, weak knockdown line) and combined *detour*^*1*^ *lt*^*Ri#2*^ knockdown midguts at 0 h RPF. The smaller gastric caeca (arrow) in *detour*^*1*^ are rescued by knockdown of *lt*. Scale bar = 200 µm. Quantitation of gastric caeca size at 0 h RPF represented as area ± SD (**p < 0.01). **f** The Atg8a immunostaining (red) of midguts at -4 h RPF shows increased Atg8a puncta in *detour*^*1*^ and *detour*^*1*^ *lt*^*Ri#2*^ with similar levels of Atg8a puncta compared to the control (*w*^*1118*^). DNA is stained by Hoechst (blue). Scale bar = 20 μm. Quantitation of Atg8a positive puncta represented as puncta/cell ± SD (*p < 0.05, **p < 0.01, ***p < 0.001). **g** The pAkt immunostaining (green) of midguts at −4 h RPF shows reduced cortical localisation (arrow) in *detour*^*1*^ compared to the combined *detour*^*1*^ with *lt*^*Ri#2*^ knockdown. DNA is stained by Hoechst (blue). Scale bar = 20 μm.

In *Drosophila*, clones depleted of HOPS subunits in the larval fat body accumulate autophagosomes, suggesting an impairment of autophagic flux^20^. To investigate the role of HOPS in regulation of autophagy in the larval midgut, we generated mosaic clones in the midgut that give rise to cells knocked down for *lt* and *dor* (marked by GFP) adjacent to control cells. Both the *lt* and the *dor* knockdown clone cells had a dramatic reduction in pmCherry-Atg8a positive vesicles compared to the neighbouring control cells at −4 h RPF (Fig. 7a, b). In addition, the size of *lt* and *dor* knockdown clone cells was significantly larger compared to the control cells (Fig. 7a, b). The cell size increase observed in the midgut was not seen in fat body cells^20^. This suggests an additional role for the HOPS complex, in addition to regulation of autophagosome fusion with the lysosome, upstream of autophagy induction.

To further examine the autophagic defect, knockdown of *lt* and *dor* was examined in the midgut. Consistent with the larger cell size, midgut morphology of *lt* and *dor* knockdown resulted in significantly larger gastric caeca compared to the control (Supplementary Fig. 6a, 0 h RPF). There was also a dramatic reduction in the level of mCherry-Atg8a positive vesicles in *lt* and *dor* knockdown midguts (+2 h RPF) (Supplementary Fig. 6b). The Atg8a staining revealed reduced puncta in *lt* knockdown with similar levels of Atg8a puncta in *dor* knockdown, however the puncta morphology was altered compared to the control (Fig. 7c; Supplementary Fig. 6c). This suggests that there is a block in autophagosome fusion with the lysosome due to the absence of mCherry-Atg8a vesicles (autolysosomes) and the presence of Atg8a immunostained positive vesicles (autophagosomes). This increase in cell and tissue size and block in autophagy suggests that HOPS is not only required for autophagosome-lysosome fusion but may play a role upstream of autophagy during midgut degradation.

Previous studies have shown that downregulation of growth signalling is essential for autophagy induction^41,52^. A marker for growth signalling in the midgut is the localization of phosphorylated Akt, required for activation of downstream signal transduction. At the onset of midgut degradation (-4h RPF) phosphorylated Akt can be detected at the cell cortex and following puparium formation (+2 h RPF) this cortical localization is greatly reduced^41^. Interestingly, the knockdown of *dor* and *lt* resulted in persistent cortical localization of phosphorylated Akt in the midgut at +2 h RPF compared to the control with decreased phosphorylated Akt at the cell cortex (Fig. 7d). Together, this data reveals a role for HOPS complex components in the downregulation of growth signalling required for autophagy induction during midgut degradation.

To investigate the in vivo significance of the interaction between detour and HOPS, we examined if there is a genetic interaction between *detour* and *lt*, as lt is a specific component of HOPS. Due to the block in autophagy induction in the *lt* knockdown, we examined another independent *lt* knockdown line to identify a sensitized genetic background. This line resulted in a decrease in *lt* transcript levels in the midgut yet did not result in a detectable midgut phenotype (Supplementary Fig. 6b). In addition, *lt* alleles were identified based on an eye pigmentation defect and consistent with the *lt*^*Ri*#2^ line resulting in a weaker knockdown, the eye phenotype of this line (*lt*^*Ri*#2^) was less severe compared to that of the strong knockdown line (*lt*^*Ri*#1^) (Supplementary Fig. 6c). The effect of simultaneous knockdown of *lt* (*lt*^*Ri#2*^) with *detour*^1^ was examined. Midgut morphology was examined during midgut degradation (0 h RPF) (Fig. 7e). The *detour*^*1*^ showed significantly reduced gastric caeca size compared to the control (Fig. 7e), as expected. Interestingly the combined knockdown of *lt* with *detour*^*1*^ increased the gastric caeca size compared to *detour*^*1*^ alone (Fig. 7e). To determine if this was due to decreased autophagy, we examined levels of Atg8a positive vesicles. Immunostaining for endogenous Atg8a revealed a significant increase in Atg8a positive staining in *detour*^*1*^, which was reduced when combined with *lt* knockdown (Fig. 7f). Given the role of lt in downregulation of growth signalling, the localisation of phosphorylated Akt was examined. This revealed that *detour* mutant had a dramatic reduction in cortical phosphorylated Akt compared to control at the same stage (Fig. 7g). Interestingly, the combined knockdown of *lt* with the *detour* mutant showed an increase in cortically localised phosphorylated Akt (Fig. 7g). Together, these findings indicate that reducing *lt* levels is sufficient to restore growth signalling thus reducing autophagy induction and midgut degradation defects in *detour* mutant. This supports an interaction between detour and HOPS complex and suggests that *detour* mutants have decreased HOPS function in the autophagy pathway resulting in delayed autolysosome formation with enhanced HOPS activity to promote downregulation of growth signalling.

### detour, ZNRF1 and ZNRF2 interact with HOPS complex

**Fig. 8:**
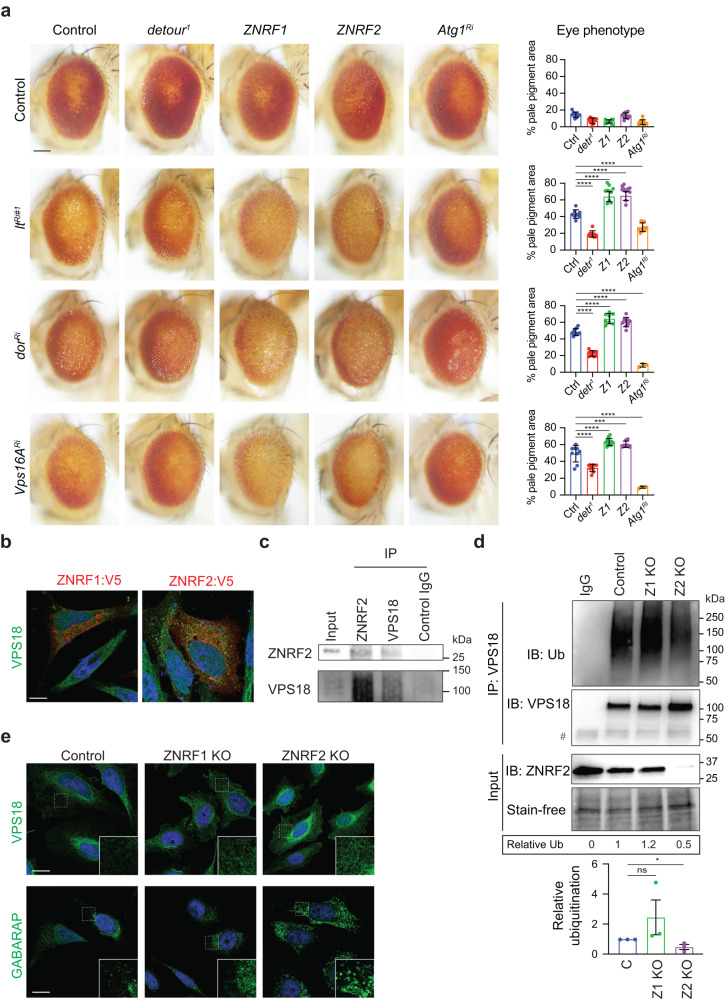
*detour*, *ZNRF1* and *ZNRF2* genetically interact with *HOPS* subunits. **a** The knockdown of *lt*^*Ri#1*^ *(GMR-GAL4, UAS-ltRi*#1/+*), dor*^*Ri*^ *(GMR-GAL4*+*, UAS-dorRi*/+*)* and *Vps16A*^*Ri*^ *(GMR-GAL4, UAS-Vsp16ARi*/+*)* in the developing eye results in a pale eye colour phenotype, with loss of pigmentation and reduced size compared to controls *(GMR-GAL4*/+*)*. The pale eye phenotype is supressed by *detour*^*1*^ and *Atg1* knockdown observed by increased red eye pigmentation and eye size. The expression of ZNRF1 and ZNRF2 enhances the *lt*^*Ri#1*^*, dor*^*Ri*^ and *Vps16A*^*Ri*^ eye phenotype. Note that *GMR-GAL4* alone results in a rough eye phenotype at 29^o^C. Scale bar = 100 μm. Quantitation of eye phenotype represented as eye area ± SD. **b** Immunostaining of HeLa cells transfected with ZNRF1:V5 and ZNRF2:V5 with VPS18 antibody (green), V5 (red), merged with nuclei stained by Hoechst (blue). Scale bar = 10 µm. **c** Total protein lysates from HeLa cells were subjected to immunoprecipitation (IP) with anti-ZNRF2, anti-VPS18 or Control IgG antibody. Proteins were separated by SDS-PAGE and immunoblotted with anti-ZNRF2 antibody or anti-VPS18 antibody. Input controls were 5% of each protein lysate. **d** Decreased ubiquitination of VPS18 in *ZNRF2* KO HeLa cells. Total protein lysate from Control, *ZNRF1* KO and *ZNRF2* KO were subjected to IP with anti-VSP18 or IgG control antibody and immunoblotted with anti-ubiquitin, anti-VPS18 and anti-ZNRF2 antibodies. Numbers represent the quantitation of Ub relative to VPS18 normalised to Control for the representative IB shown. # IgG heavy chain. Quantitation of VPS18 ubiquitination relative to control ±SEM (*p < 0.05). **e** Immunostaining of control, *ZNRF1* and *ZNRF2* KO HeLa cells with VPS18 antibody (top panel) and GABARAP (bottom panel) (green) merged with nuclei stained by Hoechst (blue). Scale bar = 10 µm. Dashed outline region is represented in the enlarged inset.

To further examine the interaction between *detour* and HOPS complex, we examined if *detour* genetically interacts with *lt, dor* and *Vps16A* in the adult eye. HOPS functions in vesicular transport of proteins to pigment granules, the specialised lysosome-related organelle responsible for eye-pigmentation. The knockdown of *lt, dor* or *Vps16A* in the developing eye (using GMR-Gal4 driver) results in loss of pigmentation compared to the wildtype control (Fig. 8a). We found that knockdown of *lt, dor* and *Vps16A* combined with *detour*^*1*^ resulted in a less severe eye phenotype, compared to knockdown of *lt, dor* and *Vps16A* alone, with an increase in eye pigmentation and reduced disorganisation of the eye pattern (Fig. 8a). Furthermore, the combined expression of ZNRF1 and ZNRF2 with the knockdown of *lt, dor* and *Vps16A* enhanced the eye phenotype with further loss of pigment (Fig. 8a). Interestingly, the reduction of autophagy by *Atg1* knockdown (or *Atg8a* mutant, DNS) was also sufficient to suppress the *lt, dor* and *Vps16A* knockdown eye phenotypes.

To assess the conserved interaction between ZNRF1 and ZNRF2 with VPS18 in mammalian cells, we examined their colocalisation in HeLa cells transfected with V5-tagged ZNRF1 and ZNRF2. This showed that cytoplasmic ZNRF2 colocalised with VPS18, to a greater extent than ZNRF1 and VPS18 (Fig. 8b). Furthermore, we were able to detect an interaction between ZNRF2 and VPS18 by co-immunoprecipitation of endogenous proteins from HeLa cells (Fig. 8c.) Importantly, the ubiquitination of endogenous VPS18 was reduced in the *ZNRF2* KO cells compared to the control (Fig. 8d). The localisation of VPS18 appeared more widespread throughout the cytoplasm in the *ZNRF1 KO* and this altered distribution was more pronounced in the *ZNRF2* KO cells (Fig. 8e). This interaction was specific for ZNRF2 as ZNRF1 did not colocalise with VPS18 and the ZNRF1 KO cells did not significantly alter VPS18 ubiquitination. There was a trend for increased ubiquitination of VPS18 in the ZNRF1 KO cells and this may be due to increased activity of ZNRF2 in the absence of ZNRF1, however this is yet to be confirmed. Together, these data suggest that the interaction between ZNRF2 and HOPS complex is conserved in mammalian cells. This also indicated that the roles of ZNRF1 and ZNRF2 in autophagy may not be redundant and may have distinct targets in the regulation of autophagy.

### detour is required for healthy ageing

**Fig. 9:**
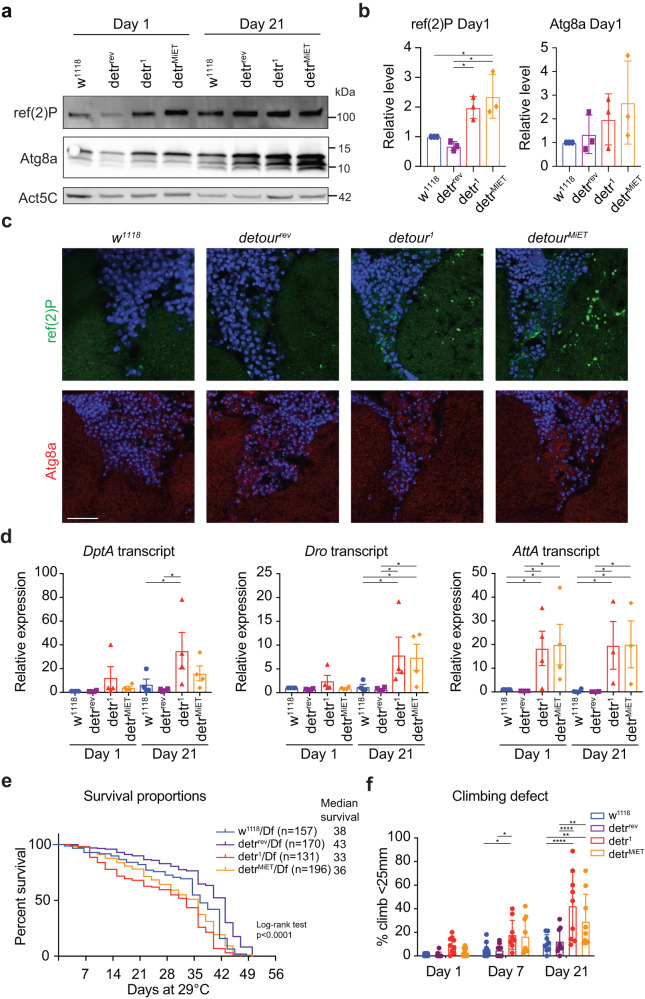
detour regulates autophagy and is required for healthy aging. **a** Immunoblot analysis of whole protein lysates from controls (*w*^*1118*^ and *detour*^*rev*^), *detour*^*1*^ and *detour*^*MiET*^ young (Day 1) and old (Day 21) adults shows endogenous levels of ref(2)P and Atg8a. Actin (Act5C) as load control. **b** Quantitation of immunoblots demonstrating a significant increase in ref(2)P protein levels in young (Day 1) *detour*^*1*^ and *detour*^*MiET*^ adults. Data presented as mean relative intensity ± SD (*n* = 3 individual repeat experiments; *p < 0.05, **p < 0.01). **c** Adult brain section of a young (0-3d old male) control, *detour*^*1*^ and *detour*^*MiET*^ showing endogenous ref(2)P and Atg8a puncta. Scale bar = 20 µm. **d** The transcript levels of selected AMP genes *Diptericin A (DptA), Drosocin (Dro)* and *Attacin-A (AttA)* from control (w^*1118*^) and *detour* mutant young (0-3 day, Day1) and old (Day 21) adults measured by qRT-PCR from total RNA with *rp49* as the reference gene. Data are from three/four independent experiments, each containing 3/5 adults per group (mean ± SEM, *p < 0.05). **e** Survival assays for female control (*w*^*1118*^*/Df* and *detour*^*rev*^*/Df*), and *detour*^*1*^*/Df* and *detour*^*MiET*^*/Df* adults. Kaplan-Meier survival assay presented as percentage of surviving population per time point (Log-rank *p* < 0.0001; Gehan-Breslow-Wilcoxon *p* < 0.0001). **f** The percent of male flies unable to climb above 25 mm (climb defect) in a cylinder after 25 s was determined every week. Data presented as mean ± SD, n ≥ 45 flies per experiment (*p < 0.05; **p < 0.01; ****p < 0.0001).

Both *detour* mutant alleles are homozygous viable and fertile, with no overt phenotype in the adults. Given the tissue-specific autophagy defects that we observed in the knockdown and mutant during development, we investigated autophagy in *detour* mutant adults. The age-related decline in autophagy in *Drosophila* adults can be monitored by the accumulation of the p62 homologue ref(2)P and Atg8a in old compared to young adults^53^. Immunoblot analysis showed that the *detour* mutant young adults (day 1) have significantly higher levels of ref(2)P protein than the control flies, and a trend towards increased Atg8a, indicating that autophagy is disrupted (Fig. 9a, b). The accumulation of ref(2)P and Atg8a protein during aging (day 21) was similar between the controls and mutants (Fig. 9a, b; Supplementary Fig. 7a). The mRNA transcript levels of *ref(*2*)P* and *Atg8a* remained similar between control and mutants in both young and aged adults (Supplementary Fig. 7b), suggesting post-transcriptional regulation. Ref(2)P is a major component of protein aggregates in flies that are defective in autophagy, and in *Drosophila* models of human neurodegenerative diseases^54^. Consistent with this, adult *detour* mutant brain sections had an increase in ref(2)P-positive structures and Atg8a puncta compared with the control brains (Fig. 9c). Interestingly, there is also an accumulation of ref(2)P in the neuropil of the *detour* mutant brains. This is similar to that seen in wild-type aged brains that accumulate ref(2)P in both neuropil and cortical regions^54^. The accumulation of ref(2)P and Atg8a in the young *detour* mutant adults indicates that autophagic flux is disrupted.

Immune activation is triggered by pathogens, and in the nervous system, it can also be triggered by autophagy^55^. In *Drosophila*, impaired autophagic flux induces an immune response, including antimicrobial peptide (AMP) expression^56–58^, which is a hallmark of many neurodegenerative diseases in humans and may contribute to decline in neuronal function^55^. Given this link, we examined whether impairment of autophagy due to *detour* ablation altered innate immunity. The levels of AMP transcripts *Diptericin A (DptA), Drosocin (Dro)* and *Attacin-A (AttA)*, under regulation of immune deficiency (IMD) pathway, were examined in the *detour* mutants and control adults. This showed a significant upregulation of AMPs in *detour* adults at both young and older ages compared to the controls (Day 1 and Day 21; Fig. 9d). The expression of AMP genes is increased in *detour* mutant flies as early as 1 day after eclosion, increasing as the flies aged 21 days. There was no difference in the expression of AMP genes following septic injury in *detour* compared to control (Supplementary Fig. 7c).

The overall health and neuronal function of *Drosophila* can be tracked by longevity assays, which reflect the deterioration in essential cellular processes such as autophagy. Longevity assays showed that *detour*^*1*^ mutant males have a reduced median lifespan compared to controls (Supplementary Fig. 7d). This was confirmed when *detour* mutant animals were crossed to the deficiency (*Df*), with a reduced median lifespan of 33 days for *detour*^*1*^*/Df* and 36 days for *detour*^*MiET*^*/Df* compared to controls with 38 days *w*^*1118*^*/Df* and 43 days *detour*^*rev*^*/Df* (Fig. 9e). The median survival of *detour* mutants was shortened as well as the maximum survival. Hence, disruption of *detour* causes dysfunctional autophagy and reduced longevity.

The age-dependent decline in neuronal function can be examined in the adult by measuring the climbing ability as it reflects the interplay between neuronal and muscular functions. *detour* mutant adults exhibited a climbing defect (<25 mm), that continues to worsen with age, with *detour* consistently displaying a more severe defect than the normal age-related defects in controls, reaching significance at day 21 (Fig. 9f). Consistent with this, the climbing ability (>160 mm), that also declines with age, was significantly reduced in aged (Day 21) *detour* mutants compared to control (Supplementary Fig. 7e). These significant defects in motor function in the *detour* mutant adults suggests that detour protects neuronal cells from neurodegeneration.

## Discussion

To identify new regulators of autophagy, we screened a collection of RNAi lines that knocked down genes encoding proteins of the ubiquitin system for defects in the degradation of the *Drosophila* larval intestine. From this we identified *Drosophila* detour, the orthologue of mammalian ZNRF1 and ZNRF2. The knockdown of *detour* resulted in premature midgut degradation and increased autophagic vesicles. Consistent with this, increased Atg8a levels and smaller cell size was observed in mosaic clone cells ablated for *detour* in the midgut. We generated *detour* mutant lines that also displayed smaller midgut size with increased Atg8a-positive autophagic vesicles. There was also an increase in Atg5-positive vesicles suggesting an increase in early-stage phagophores and an increased ref(2)P suggesting a decrease in cargo transport to the lysosome. Consistent with this, while there was an increase in phagophores and autophagosomes, there was no corresponding increase in autolysosome. Ultrastructural analysis also revealed an increase in both phagophores and autophagosomes. Furthermore, in the midguts ablated for *detour*, the Atg8a positive puncta appeared to be distinct from Rab7 positive vesicles. This suggests that there is an accumulation of phagophores and autophagosomes and a delay in the formation of autolysosomes in *detour* mutants. In the absence of components of two ubiquitin-like conjugation systems, the E3-like Atg5 or E2-like Atg3, isolation membranes form and persist as open-ended vesicles^59–61^. However, these vesicles eventually form closed autophagosomes that fuse with lysosomes, indicating that the rate of transition from phagophore to autophagosome is delayed in ATG-conjugation deficient cells^62^. However, as *detour* mutants accumulate both phagophores and autophagosomes without an increase in autolysosomes, this suggests that fusion with the lysosome is delayed. The role of detour in autophagy is further supported by the genetic interactions identified between *Drosophila* Atg1 and *detour*. Taken together, this supports a role for detour as a positive autophagy regulator and that *detour* mutants accumulate autophagic vesicles due to a delay in autolysosome formation.

The overexpression of detour, ZNRF1 or ZNRF2 in *Drosophila* midguts led to an increase in autophagic vesicle size, with a decrease in ref(2)P. This is consistent with an increase in autolysosome and the increase in Rab7 and Atg8a positive puncta. This suggests that autophagic vesicle maturation is enhanced following overexpression of detour, ZNRF1 or ZNRF2, leading to the accumulation of late stage autophagosomes and/or autolysosomes. The role of detour, ZNRF1 and ZNRF2 in modulation of autophagy is supported by the genetic interactions between detour, ZNRF1 or ZNRF2 and Atg1. To further establish if the function of detour is conserved in mammals, we generated *ZNRF1* and *ZNRF2* deficient HeLa cells. The *ZNRF1* and *ZNRF2* KO cells had increased LC3 lipidation with *ZNRF2* KO cell also having increased p62. Conversely, their overexpression in HeLa cells was sufficient to reduce p62 levels. This suggests that ZNRF1 and ZNRF2 are required to maintain basal autophagy under normal growth conditions, similar to *Drosophila* detour. These findings suggest that ubiquitin ligases detour, ZNRF1 or ZNRF2 promote the fusion of autophagosomes with lysosomes, leading to increased autophagic vesicles due to the accumulation of enlarged autophagosomes and/or autolysosome.

We identified HOPS complex subunits, dor/VPS18, Vps16A and lt/VPS41 as interactors of detour. Our studies have focused on HOPS due to the interaction with the HOPS specific subunit, lt. It is also important to note that as dor and Vps16A are shared with CORVET the activity of this complex could be examined in future studies. The HOPS complex coordinates the fusion between autophagosomes and lysosomes. The autophagy defects observed following modulation of detour are consistent with that due to the altered HOPS complex activity, that similarly results in impaired autophagic flux. We propose that detour is required for autophagosome maturation and/or autophagosome-lysosome fusion due to its role in regulating HOPS complex. Furthermore, our study identified an additional role for HOPS complex in downregulation of growth signalling in the *Drosophila* larval midgut. Our results suggest that in the absence of detour there is a decrease in autophagosome fusion with the lysosome as well as an increase in autophagy induction. This may be due to premature downregulation of growth signalling as HOPS is now recruited to growth signalling pathway and despite the increased autophagy induction flux is delayed as there in no increase in autolysosomes. This suggests that in the absence of detour, HOPS is no longer tethered to autophagy pathway but is recruited to growth signalling pathway resulting in enhanced downregulation of phosphorylated Akt. The ablation of *detour*, *ZNRF1* or *ZNRF2* may prevent the maturation of autophagic vesicles due to defects in HOPS-dependent membrane fusion events that delay autophagic cargo delivery to lysosomes. The overexpression of detour, ZNRF1 or ZNRF2 may enhance vesicle fusion between autophagic vesicles and lysosomes, leading to an accumulation of autolysosomes. We propose that membrane-associated ubiquitin ligases detour, ZNRF1 and ZNRF2, through an interaction with HOPS complex, regulate the rate of the delivery of autophagic cargo to the lysosome.

ZNRF1 and ZNRF2 are closely related members of the RING superfamily of ubiquitin ligases with detour the single orthologue in *Drosophila*. Their E3 RING domains can recruit and tether E2 ubiquitin-conjugating enzyme complexes to regulate the formation of Lys63-ubiquitin or Lys48-ubiquitin linkages^35,37,38,48,49,63,64^. This Lys63-linked polyubiquitination of proteins can promote membrane trafficking and lysosomal degradation. Both ZNRF1 and ZNRF2 are localised to vesicle membranes by N-myristoylation and this is important for ZNRF2 localisation and interaction with mTORC1^35,48^. The *S. cerevisiae* homologue, the transmembrane RING domain E3 Pib1, also associates with the target membrane (via interactions with phosphoinositides) and localises to endosomes and vacuoles with a role in vacuolar sorting and multivesicular body pathway^65,66^. As detour also contains a conserved motif for N-myristoylation, this suggests an important evolutionarily conserved membrane-bound E3 family that regulates vesicle fusion events important to maintain autophagic flux.

ZNRF1 and ZNRF2 are present at the neuromuscular junction and strongly expressed in mouse neurons^36,37^, and ZNRF1 promotes axonal degeneration^38^. ZNRF2 has been shown to have a protective role in cerebral ischaemia/reperfusion injury in rats^67^. Our findings show that loss of *detour* results in dysfunctional autophagy, with increased expression of AMP genes, motor function defects and decreased lifespan. This indicates that detour functions more broadly in maintaining autophagic flux and is important for preventing premature neurodegeneration and promoting healthy aging. Effective autophagic-lysosomal clearance of dysfunctional proteins is particularly important in neuronal cells to maintain homeostasis to prevent neurodegenerative effects throughout their lifespan. The identification of VPS41 mutations in patients with a severe neurological disorder, highlights the important function of HOPS-dependent delivery of autophagic cargo to lysosomes^23^. This suggests that ZNRF2 may act to regulate HOPS dependent transport/fusion which will be important for future studies. As there was previously no direct evidence linking ZNRF1 or ZNRF2 to autophagy, this new information raises important questions as to their functions in neurons, particularly under pathogenic conditions.

Based on our findings we propose a model whereby detour promotes the recruitment of HOPS to the autophagy pathway and perturbations to detour level result in an altered balance of HOPS complex tethering to other organelle localisations. This is supported by both cell biology and genetic interactions. Induction of autophagy can occur, yet the downstream maturation and lysosomal fusion are altered in response to changes in detour, ZNRF1 and ZNRF2 levels. In the midgut, detour promotes the localisation of HOPS to the autophagy pathway to regulate autophagosome fusion with the lysosome. When detour levels are reduced this alters the pool of HOPS acting in the autophagy pathway and facilitates HOPS to act in growth signalling pathway. In the eye, tethering HOPS to the autophagy pathway by ZNRF1 and ZNRF2 overexpression leads to a reduced pool of HOPS components to act in the eye-pigmentation pathway, resulting in the enhanced eye phenotype. Conversely, in the absence of detour HOPS is no longer recruited to autophagy and increases the pool that can act in eye pigmentation. Similarly, reducing autophagy by *Atg1* knockdown, is sufficient to supress the eye pigmentation defect. This suggests that reduced autophagy and hence reduced role of HOPS complex, results in an increased pool of HOPS to act in the eye pigmentation pathway. Alternative models are also possible, including that autophagy regulation by detour may be due to the alteration to growth signalling as seen in the maintained pAKT in the detour mutants and given the role of ZNRF2 on mTOR^35^.

Adaptors play important roles in the recruitment of protein complex to specific cytoplasmic compartments. The Valosin Containing Protein (VCP) is an AAA+ ATPase that localises to several cellular compartments including lysosomes, with mutations linked to several degenerative diseases^68^. The VCP-binding co-factor (SVIP) recruits VCP to lysosomes and is essential for autophagosomal-lysosomal fusion^69^. Mutation in SVIP result in degenerative defects, including declining locomotor activity and decreased life span^69^. The importance of subcellular localisation of autophagy regulators is also seen in *C. elegans*, where the RING ubiquitin ligase RPM-1, a PAM/Highwire/RPM-1 protein family homologous to mammalian Phr1/MYCBP2, spatially restricts UNC-51(ULK) degradation in neurons to inhibit autophagy^70^. In the absence of *rpm-1*, excessive autophagy results in axonal and behavioural defects^70^. Recently, variants in MYCBP2 have been identified in patients with a neurodevelopmental disorder^71^. Functional analysis of the variants using *C. elegans* model suggests that they increase axonal autophagosome formation and have defects in axon development and behaviour^71^. We propose that detour acts as a regulator of HOPS complex by promoting ubiquitination of HOPS subunits to tether the complex to the autophagy pathway. This highlights the critical role of tethering protein complexes to the appropriated compartment for the regulation of autophagic flux and that defects can have pathological consequences.

With dysregulation of autophagic flux identified in the pathology of many human diseases, including neurodegeneration and cancer, it is important to understand how this contributes to pathogenesis. The identification of detour in *Drosophila* and mammalian homologues ZNRF1 and ZNRF2 has provided the opportunity to define the role of the evolutionarily conserved membrane-associated RING E3 in autophagy in vivo. This study uncovers a function of the E3 detour, that interacts with HOPS complex and regulates autophagosome biogenesis.

## Methods

### *Drosophila* strains

The midgut driver *Mex-GAL4* was obtained from Richard Burke (Monash University, Vic., Australia). The following stocks were from the Bloomington *Drosophila* Stock Center (Bloomington, IN, USA): *w*^*1118*^ *Mi{GFP[E.3xP3]=ET1}CG14435*^*MB05816*^ (BL25464)*, w*^*1118*^*; sna*^*Sco*^*/SM6a, P{w[+mC]=hsILMiT}2.4* (BL24613*), w*^*1118*^ (BL3605), *w*^*1118*^ *Df(1)BSC297/Binsinscy* (BL23681), *UAS-ZNRF1:HA* (BL79151), *UAS-ZRNF2* (BL86229), *GMR-GAL4* (BL1104), *UAS-lt* RNAi (BL34871), *UAS-Vps16A* RNAi (BL38271), *UAS-eGFP:Atg5* (BL59848), *UAS-GFP:LAMP1* (BL42714). The knockdown lines for *CG14435R*-1 (270R1), *UAS-dor* RNAi (3093R-2) and UAS-*lt* RNAi (18028R-2) were obtained from NIG-FLY, and *CG14435{GD8324}* (v17600), *UAS-Atg1* RNAi from Vienna Drosophila RNAi Center. The *NP1-GAL4* (112001) and *UAS-Atg1*^GS10797^ (contains UAS regulatory sequences inserted upstream of the endogenous *Atg1* gene) lines were obtained from Kyoto Drosophila Genetic Resource Center. The *pmCherry-Atg8a* line expresses mCherry-tagged Atg8a from the endogenous *Atg8a* promoter, was used as a marker of autophagy^41^, and to generate mosaic clones *hsFLP; pmCherry-Atg8a; Act* > *CD2* > *GAL4, UAS-nlsGFP/TM6B* line was used (from E. Baehrecke). Transgenic *UAS-detour-EGFP* lines were generated by BestGene Inc (CA, USA). The control was *w*^*1118*^ crossed to *Mex-GAL4*, or relevant driver line. The knockdown quantitation of the RNA*i* lines was determined by qRT-PCR from a minimum of 3/sample in triplicate. All flies were maintained and crossed performed at 25 °C unless otherwise stated, on *Drosophila* media [18.75% compressed yeast, 10% treacle, 10% polenta, 2.5% tegosept (10% parahydroxybenzoate in ethanol), 1.5% acid mix (47% proionic acid, 4.7% orthophosphoric acid) and 1% w/v agar].

### Generation of the *detour*^*1*^ mutant

An additional *detour* mutant allele was generated by mobilisation of a 7.5 kb *minos* transposable element within *detour*^*Mi*^ (*w*^*1118*^ *Mi{ET1}CG14435*^*MB05816*^, marked by EGFP under *Pax6* promoter) by crossing to the *hsILMiT* transposase to produce lines with imprecise excisions affecting expression of the gene^72^. The mutant lines were identified by loss of EGFP expression in the eye and were crossed to *w*^*1118*^ for up to 6 generations to eliminate any off-target effects during this process. An excision event was identified in detour EGFP negative flies over *Df(1)BSC297* from single flies. The genomic deletions were determined by sequencing with specific primers spanning the insertion region. Briefly, genomic DNA was prepared from the *detour*^*1*^ mutant and used as the template for PCR. Gel-purified PCR products were sequenced at SA Path. Sequences from the *CG4435*^*1*^ mutant were compared to those from the parent X chromosome. A deletion was identified within intron 3 of putative *detour* transcript removing exon 4 and 5, which was designated as *detour* mutation 1 (*w*^*1118*^*, detour*^*1*^) (Fig. 2a). The *detour*^*rev*^ resulted from a precise excision.

### Generation of constructs

Full-length detour, lt, and V5 control gBlock® gene fragments (IDT) were cloned into the pENTR™/D-TOPO® or pENTR™/SD/D-TOPO® using the pENTR™ Directional TOPO® Cloning Kit (Invitrogen) according to the manufacturer’s instructions. As detour contains a conserved motif for N-myristoylation, C-terminally tagged constructs were generated. To generate construct for expression in S2 cells, TOPO-detour and TOPO-V5 were used to recombine inserts into the C-term EGFP tagged vector with Actin5C promoter, pAWG, destination vector (*Drosophila* Genomics Resource Center, #1072). A transformation construct for the UAS-detour-EGFP transgenic line was generated by insertion of TOPO-detour insert into in the C-term EGFP tagged P-element transformation vector, pTWG (Carnegie Institution for Science, Drosophila Gateway™ Vector Collection). Reactions were performed using the Gateway™ LR Clonase™ II Enzyme Mix (Invitrogen) according to the manufacturer’s instructions. Full length dor, lt and Vps16A constructs were generated by Gateway cloning into the pENTR™/D-TOPO® (Invitrogen) according to the manufacturer’s instructions. The TOPO-dor, TOPO-lt and TOPO-Vps16A were used to recombine inserts into the appropriate tagged vector with Actin5C promoter, to generate pAFW-dor (FLAG:dor), pAMW-Vps16a (Myc:Vps16A), pAFW-Vps16A (Flag:Vps16A), pAHW-lt (HA:lt) and pAFW-lt (Flag:lt). Full length detour (TOTO-detour) was inserted in pIB-V5-His (RfB). Full-length ZNRF1 and ZNRF2 gBlock® gene fragment (IDT) were cloned into the pENTR™/D-TOPO® (Invitrogen) according to the manufacturer’s instructions and used to recombine into pcDNA3.2 to generate ZNRF1:V5 and ZNRF2:V5 constructs. Reactions were performed using the Gateway™ LR Clonase™ II Enzyme Mix (Invitrogen) according to the manufacturer’s instructions. The ubiquitin expression construct pIE4-HA-Ub was as described^73^. gBlock® for detour, lt, ZNRF2 and V5 were used for Gateway® cloning (Supplementary Table 1). Cloning primers (IDT) use are as follows: *dor* F 5’-caccATGGACACGTCTATGCCTAACC; R 5’-GTCTGAACGACGGTGGTAGC, *lt* F 5’-caccATGGCTAAAGCGTTGCCGCTC; R 5’-CGGGGTAACAGTTATGATGTCGC, *Vps16A* F 5’-caccATGCCTATCATGTACAACACGGGG; R 5’-AGCTTAAGAGTGCTATTCGTATAGAC.

### Larval staging and midgut morphology analysis

Wandering third instar larvae raised on standard media supplemented with 0.05% bromophenol blue were transferred to a petri dish with moist Whatmann paper to monitor for gut clearance as visualized by loss of blue in the gut (-4 h RPF)^74^. For morphological analysis, a minimum of 10 midguts were dissected in 1xPBS from appropriately aged animals fixed in 4% paraformaldehyde v/v in 1xPBS^75^. Images were acquired using a stereo microscope (SZ61, Olympus, Tokyo, Japan) equipped with a 2× auxiliary objective (110AL 2x, Olympus) and digital camera (DP21, Olympus). To determine gastric caeca size, the gastric caeca was outlined with the magnetic lasso tool in Adobe Photoshop CS6 (Adobe, San Jose, CA, USA) and the number of pixels in this area was measured using the histogram function. The mean and SD. of gastric caeca size were calculated using Prism (GraphPad Software).

### Live mCherry, eGFP and LysoTracker imaging

For live imaging of mCherry-Atg8a or eGFP:Atg5, a minimum of 10 midguts were dissected from appropriately staged animals in 1xPBS with Hoechst 33342 (2 μg/ml, Sigma-Aldrich) to stain DNA and imaged immediately without fixation using a Zeiss confocal (LSM 800, Carl Zeiss Microscopy, Jena, Germany). The images were quantitated using ImageJ (Bethesda, MD, USA) to count puncta with a size >2 pixels and represented as the average puncta per cell. For LysoTracker staining, midguts were dissected in 1xPBS with 1 μM LysoTracker Green DND26 (1:1000; Invitrogen Molecular Probes, L7526) and Hoechst 33342.

### Immunostaining *Drosophila* tissue

Heads from animals of the required age were dissected and fixed with 4% v/v paraformaldehyde in 1xPBS at room temperature for 60 mins then washed for 60 mins in 1xPBTw (PBS + 0.1% tween) prior to overnight incubation in 30% sucrose in PBS on nutator at 4 °C. Tissues were embedded in OCT and stored at -80 until 14 micron sections cut. Tissue sections were then blocked with 1xPBS + 0.3% triton X + 1% BSA. For midgut tissue, animals of the required stages were dissected in 1xPBS and fixed with 4% v/v paraformaldehyde in 1xPBS at room temperature for 45 mins. Tissues were then washed 3 x 10 min 1xPBTx (PBS + 0.1% triton X) and blocked with 1xPBTx + 1% BSA at room temperature for 1 h. Samples were incubated with primary antibody in blocking buffer overnight at 4 °C. Primary antibodies were: rabbit anti-ref(2)P 1:500 (Abcam ab178440), rabbit anti-GABARAP 1:200 (referred to as Atg8a; Abcam ab109364), mouse anti-Rab7 1:10 (DSHB), rabbit anti-phospho-Akt 1:200 (Cell Signalling 4054) and goat anti-GFP 1:500 (Rockford 600-101-215). Following 4 × 30 min washes in 1xPBTx (PBS + 0.1% triton X), samples were incubated with secondary antibodies at room temperature for 1-2 h. Secondary antibodies: anti-rabbit Alexa-FLUOR 488, anti-rabbit Alexa-FLUOR 555, anti-mouse Alexa-FLUOR 488 or anti-mouse Alexa-FLUOR 647 (1:200, Molecular Probes, Eugene, CA, USA). Samples were washed 3 × 10 min in 1xPBTx, stained with Hoechst 33342 (2 µg/µL) for 1 min and mounted in ProLong® Gold Antifade (Thermo Scientific) or 80% glycerol/PBS and imaged on a Zeiss LSM 800 confocal microscope (Carl Zeiss Microscopy, Jena, Germany).

### Confocal microscopy

Confocal images were obtained using a Carl Zeiss LSM 800 Axio Observer 7 confocal microscope with 405 nm (5 mW), 488 nm (10 mW), 561 nm (10 mW) and 640 nm (5 mW) lasers visualized with PlanApo 40x/1.3 or 63x/1.4 Oil DIC objectives (Carl Zeiss Microscopy, Jena, Germany). Images captured by Zen 2011 (Black Edition) software (Carl Zeiss Microscopy) were exported into Photoshop (Adobe, San Jose, CA, USA).

### Transmission electron microscopy

Midguts from appropriately staged larvae were dissected in 1xPBS then fixed in 1.25% glutaraldehyde, 4% sucrose, 4% paraformaldehyde in PBS for 30 min at room temperature. Samples were then washed with 4% sucrose in PBS, post-fixed in 1% osmium tetroxide for 1 h, dehydrated, treated with propylene oxide, and infiltrated for embedding in resin^75^. Ultrathin sections were cut on grids, stained for 2 min with 4% uranyl acetate in 25% ethanol and 2 min in Reynold’s lead citrate before examination using Tecnai G2 Spirit TEM (Adelaide Microscopy).

### Lifespan assay

Newly eclosed flies (D0-3) were collected in groups of 20 per vial with a minimum of four replicates per genotype and maintained at 29 °C. They were transferred to fresh medium every 2–3 days and deaths recorded. Each experiment was repeated at least two times. Kaplan–Meier curves and statistical analysis was performed according to Prism survival function, with a log-rank Mantel–Cox test to determine statistical significance.

### Climbing assay

Locomotor decline during aging was monitored by conducting climbing assay. Briefly, without anaesthetization, 2 × 15 male flies of respective genotype were transferred into 500 mL measuring cylinders, each containing 15 flies. The top of the cylinder was sealed with parafilm to prevent flies escaping. The cylinder was gently tapped three times to bring the flies down to the bottom and the number of flies that climb up within each predefined area of the cylinder (<50 mL = 25 mm, >300 mL = 160 mm) after 25 s was recorded. This assay was performed weekly from day 1 post-eclosion to day 21 age. Three replicates were conducted for each cohort (*n* = 15) of flies and were allowed 2 min reset before tapping down and 1 min between each assay.

### S2 cell culture and transfection

S2 cells were cultured in complete Schneider’s medium in a 28 °C humidified incubator (Sanyo) and passaged twice a week at 1:2–1:4 dilutions. For each transfection, 52.5 µL of Cellfectin® II transfection reagent (Invitrogen), 7 µg of plasmid DNA and 42 µL of PLUS™ reagent (Invitrogen) were diluted in 550 µL of Schneider’s medium and incubated for 5 min at room temperature. The diluted plasmid DNA and diluted Cellfectin® II reagents were combined and incubated for 20 min at room temperature. The cell medium was replaced with 5 mL of plasmid DNA-Cellfectin® II mixture resuspended in Schneider’s medium. Cells were incubated in a 28 °C humidified incubator (Sanyo) for 4 h before adding 5 mL of Schneider’s medium supplemented with 20% FCS. Transfected cells were harvested for protein extraction 48 h post-transfection.

### ZNRF1-V5 and ZNRF2-V5 transfections

4 × 10^5^ HeLa cells were transfected with 2 μg of plasmid using Lipofectamine 3000 according to manufacturer’s instructions. 24 h post-transfection, cells were lysed in 30 µL of ice-cold NP-40-containing lysis buffer and 20 µL processed for immunoblotting as below.

### Immunostaining cells

HeLa cells were grown in DMEM media (Gibco) supplemented with 10% FBS, 2mM L-glutamine, 100 µM penicillin/streptomycin and HEPES. Cells were plated on glass coverslips overnight at 2 × 10^5^ cells/mL and fixed for 20 min in 4% v/v paraformaldehyde in 1xPBS at room temperature, then incubated with 1xPBTx for 10 min. Cells were washed 3 times in 1xPBS and blocked for 30 min with blocking buffer (1% BSA, 1% goat serum in PBS). The blocking buffer was removed and primary antibodies, anti-Sequestosome 1 (SQSTM1/p62) (Abnova H00008878-M01 Mouse; 1:200), anti-GABARAPs (Abcam ab109364 Rabbit; 1:200) or anti-V5 (Abcam 27671 Mouse 1:200), incubated at room temperature for 2 h. Cells were then washed 3 times in 1xPBS. The AlexaFluor-488 (1:200) secondary antibody was incubated at room temperature for 1 h. Cells were then washed 3 times in 1xPBS and counterstained with Hoechst 33342 and mounted in Prolong Gold Antifade. For chloroquine treatment, 200 µM of chloroquine was added for 4 h prior to staining.

### LysoTracker and magic red staining

HeLa cells were grown as for immunostaining. For LysoTracker staining, cells were incubated in 1xPBS with 1 μM LysoTracker Green DND26 (1:1000; Invitrogen Molecular Probes, L7526 respectively) and Hoechst 33342 for 5 min then washed in 1xPBS and imaged immediately. The Magic Red staining was performed according to manufactures protocol, Cathepsin L Assay Kit (Magic Red) (ab270774). For fluorescence plate reader analysis of Magic Red, cells were plated in triplicate in a 96-well flat bottom transparent plate according to manufactures protocol and fluorescence readings were recorded using a microplate reader Spark 10 M with SparkControl Magellan (Tecan Group Ltd., Männedorf, Switzerland).

### Generation of *ZNRF1* and *ZNRF2* KO HeLa cell lines

*ZNRF1* and *ZNRF2* KO HeLa cell lines were generated using CRISPR-Cas9 technology^76^. The small guide RNAs (sgRNAs) targeting a region in exon 1 of human ZNRF1 and ZNRF2, respectively, were cloned into the pSpCas9(BB)-2A-Puro (PX459) vector plasmid (a gift from Feng Zhang; Addgene plasmid # 48139; http://n2t.net/addgene:48139; RRID:Addgene_48139). The following sgRNA oligos (IDT) were used:

ZNRF1: Sense 5’-CACCGCTCCTGGTAACCATTGCCAT

Anti-sense 5’-AAACATGGCAATGGTTACCAGGAGC,

ZNRF2: Sense 5’-CACCGAGCCCGAGTACGCGCGCGTG

Anti-sense 5’-AAACCACGCGCGCGTACTCGGGCTC

The plasmid was transfected using Lipofectamine 3000 reagent (Invitrogen), with an empty PX459 vector serving as a control to generate wild type clones. After 24 h, media was replaced and supplemented with 2.5 μg/mL puromycin (Sigma-Aldrich) for a further 24 h to select for transfected cells. Surviving cells were passaged at low seeding density and single colonies selected and propagated. To screen candidate single clones for both *ZNRF1* KO (B8), *ZNRF2* KO (D9) and control wild type (W6) (empty PX459 vector), the sgRNA-bound area was amplified by PCR with following primers:

ZNRF1 F 5’-TGCTGCTGAGAAGTGGGGGAG; R 5’-CTAGTTTCCAGACGGTAGGTATC, ZNRF2 F 5’-ACCAGAACCGAAACCAGCG; R 5’-GCTG GGCACCTGAGCC.

The PCR products were run on 2% agarose gel and purified with QIAquick Gel extraction Kit (Qiagen) according to the manufacturer’s instruction and were subjected to DNA sequencing. DNA sequencing results were used for insertion and deletion analysis in the targeted gene area by TIDE software (http://tide.nki.nl) and alignment by ClustalW software (https://www.genome.jp/tools-bin/clustalw). Loss of *ZNRF1* and *ZNRF2* expression was validated by qPCR and /or Western blot.

### Quantitative real time PCR (qRT-PCR)

Total RNA was isolated from cells, whole flies or midguts dissected from appropriately staged animals using TRIzol® reagent (Life Technologies, Carlsbad, CA) according to the manufacturer’s instructions. cDNA synthesis was performed using the High Capacity cDNA Reverse Transcription Kit (Applied Biosciences) with 1 µg of RNA, random primers and RNaseOUT™ Recombinant Ribonuclease Inhibitor (Thermo Fisher) according to the manufacturer’s instructions. qRT-PCR was performed using KAPA SYBR® FAST according to manufacturer’s instructions on a Rotor-Gene Q (Qiagen, Valencia, CA, USA) controlled with Rotor-Gene software. Reactions were performed in triplicate and normalised gene expression was calculated relative to that of control gene (*rp49*, also known as *rpL32*, for *Drosophila* and *β-actin* for human) using standard curves in Q-Gene software. Primers used for qRT-PCR from IDT, except *detour* and *dor* from GeneWorks (Supplementary Table 2).

### Protein extraction

Transfected S2 cells were centrifuged at 2000 rpm for 2 min at room temperature and resuspended in 1 mL of 1xPBS. These pellets were either resuspension in 500 µL of ice-cold NP-40-containing lysis buffer containing Halt™ complete protease/phosphatase single-use inhibitor cocktail (Thermo Scientific) and 0.5 M EDTA (Thermo Scientific). Protein was then extracted by probe sonication using an ultrasonic liquid processor (Vibra-Cell™ VCX 130) equipped with a 1/8th inch stepped microtip (Sonics) for a total sonication time of 30 s (Amp: 1 50%, ON:10 s, OFF: 10 s, ON: 10 s). Lysates were centrifuged at 13,200 rpm for 10 min at 4 °C to pellet debris and the remaining lysates were transferred to new microcentrifuge tubes.

### GFP Immunoprecipitation

150 µL of GFP-Trap®_MA bead slurry (Chromotek) per immunoprecipitation was equilibrated by washing three times with 1 mL of ice-cold NP-40-containing lysis buffer. 15 µL and 20 µL of lysate were reserved as the input for mass-spectrometry (MS) and immunoblot analysis respectively, while the remaining lysates were added to the equilibrated beads and left overnight at 4 °C on a nutator. The beads were then briefly centrifuged in a microcentrifuge and the supernatant was collected for immunoblot analysis. The beads were washed three times in 1 mL of ice-cold NP-40-containing lysis buffer followed by a final wash in 1 mL of lysis buffer without NP-40 to remove the detergent. Any residual lysis buffer was removed by briefly centrifuging in a microcentrifuge. Proteins were then eluted by adding 50 µL of 0.2 M glycine (pH 2.5) for 2 min with constant mixing by gently flicking the tubes. Eluates were transferred to new microcentrifuge tubes and neutralised with 5 µL of 1 M Tris-HCl (pH 10.4). The elution and neutralisation steps were performed three times.

### Endogenous immunoprecipitation

HeLa cells harvested from a confluent T75 flask were lysed in 500 µL ice-cold NP-40-containing lysis buffer. 50 µL of lysate was reserved as the input, and the remaining lysates were precleared for 30 min at 4 °C with magnetic protein G-Sepharose (Amersham Biosciences). Precleared samples were incubated with 10 μl of magnetic protein G-Sepharose beads and 1 µL of either rabbit anti-ZNRF2 (Novus Biologicals NBP1-28715), rabbit anti-VPS18 (Abclonal A16654) or rabbit anti-IgG. The protein-bound beads were then washed twice in lysis buffer and once in PBS, boiled at 95 °C for 5 min and run on 10% SDS-PAGE gels for immunoblotting as below.

For the detection VPS18 ubiquitination, parental (W6), ZNRF1 KO (B8) and ZNRF2 KO (D9) HeLa cells were incubated with 10 µM MG132 (Sigma-Aldrich) for 4 h. The cells were lysed in 100 µl cell lysis buffer (2% SDS, 150 mM NaCl, 10 mM Tris-HCl, Ph 8.0) in a 1.5 ml tube, followed by incubation at 95 °C for 10 min. Next, 900 µl dilution buffer (10 mM Tris-HCl, 150 mM NaCl, 2 mM EDTA, 1% Triton X-100, Ph 8.0) was added, followed by sonication. The diluted samples were spun at 15,000 rpm for 20 min at 4 °C. The supernatant was mixed with protein A/G beads and rabbit anti-VPS18 antibody (Abclonal A17563) in a 1.5 ml tube and incubated overnight at 4 °C with rotation. After four times wash as described above, the beads were boiled in 40 µl 2 x SDS loading buffer for immunoblotting.

### Detection of protein ubiquitination in cultured S2 cells

Transfected S2 cells were lysed with 100 µl cell lysis buffer (2% SDS, 150 mM NaCl, 10 mM Tris-HCl, pH 8.0) in a 1.5 ml tube and heated for 10 min at 100 °C, followed by sonication. After sonication, 900 µl dilution buffer (10 mM Tris-HCl, pH 8.0, 150 mM NaCl, 2 mM EDTA, 1% Triton) was added and the diluted samples were spun at 15,000 rpm for 20 min at 4 °C. The supernatant was transferred to a new tube. Protein G/A agarose magnet beads (Thermo Fischer) were equilibrated with dilution buffer. The cell lysate, protein G/A beads and rabbit anti-HA antibody were mixed and incubated overnight at 4 °C with rotation. The tubes were placed on a magnet stand and the supernatant was aspirated. The protein G/A beads were washed with dilution buffer four times. After the last wash, the beads were boiled with 2 x SDS loading buffer and subjected to immunoblotting.

### Immunoblotting

To denature proteins for SDS-PAGE, 10 µL of protein lysates were boiled in protein loading buffer for 4 min and centrifuged at 13,000 rpm for 1 min. Prepared samples were run on a 15-well 4-15% Mini-PROTEAN® TGX™ Stain-Free™ pre-cast polyacrylamide gel (Bio-Rad, Hercules, CA, USA) for 1 h at 110 V in SDS-PAGE running buffer (250 mM Tris, 192 mM glycine, 0.06% SDS). The proteins were transferred onto PVDF or nitrocellulose membrane (Bio-Rad) using a Trans-Blot Turbo (Bio-Rad), for 30 min at 25 V in transfer buffer (25 mM Tris, 192 mM glycine, 20% methanol, 0.05% SDS) according to the manufacturer’s instructions. Membranes were blocked with 5% w/v skim milk (Diploma) in TBST blocking buffer [TBST (20 mM Tris, 150 mM NaCl pH 7.4, 0.05% Tween- 20)], for 1 h at room temperature. Membranes were incubated overnight with anti-GFP (Rockford 600-101-215 Goat 1:500), anti-HA (Cell Signalling CST.2367 S (6E2) Mouse 1:500), anti-FLAG (Sigma F1804 Mouse 1:500), anti-Myc (NEB 2276 S (9B11) Mouse 1:500), anti-Microtubule-associated protein 1 light chain 3 (LC3B) (Novus Biologicals 100-2220 Rabbit 1:200), anti-Sequestosome 1 (SQSTM1/p62) (Abnova H00008878-M01 Mouse 1:200), anti-ZNRF2 (Novus NBP1-28715 Rabbit 1:500), anti-V5 (Abcam ab27671 Mouse 1:500), anti-VPS18 (Abclonal A16654 Rabbit 1:500), anti-VPS18 (Abclonal A17563, Rabbit 1:1000), anti-Ubiquitin-horseradish peroxidase (HRP) (Santa Cruz sc-8017 Mouse 1:500), anti-Cathepsin L/MEP (Abcam ab58991 Rabbit 1:500) or anti-Act5C/ACTB/β-actin (Sigma Aldrich A1978 Mouse), diluted in blocking buffer at 4 °C with rocking. Membranes were washed 4×15 min with TBST before incubation with anti-goat horseradish peroxidase (HRP) (1:20,000), also diluted in blocking buffer, for 1 h at room temperature with rocking. Membranes were then washed 4×10 min with TBST then developed using ECL™ Prime Western Blotting Detection Reagent (GE Healthcare) and imaged using the ImageQuant LAS 4000 and Typhoon FLA 9000 (GE Healthcare).

### Mass spectrometry

GFP-Trap protein samples were resuspended in 6 M Urea, 100 mM DTT and 100 mM Tris-HCl pH 7.0, and subjected to protein digestion using a FASP column (Expedeon Inc.)^77^. Peptides were lyophilised to dryness, resuspended in 2% ACN and 1% FA before injection and separation by reversed-phase liquid chromatography on an M-class ultra-high performance liquid chromatography (UHPLC) system (Waters) using a 250 mm × 75 μm column (1.6 μm C18, packed emitter tip; Ion Opticks) with a linear 90-min gradient at a flow rate of 400 nL/min from 98% solvent A (0.1% FA in Milli-Q water) to 35% solvent B (0.1% FA, 99.9% ACN). The nano-UHPLC was coupled on-line to an Impact II mass spectrometer equipped with a CaptiveSpray ionization source (Bruker Daltonics) and column oven at 40 °C (Sonation). The Impact II was operated in a data-dependent mode using a 1.5 s cycle time, switching automatically between one full-scan (4 Hz) and subsequent MS/MS scans for the remaining time with spectra rate determined using peptide intensity. The raw files were analysed using the MaxQuant software^78,79^ version 1.5.8. The database search was performed using *D. melanogaster* protein sequences obtained from UniProt.

### Bioinformatics

The obtained Universal Protein Knowledgebase (UniProt v2.16; http://www.uniprot.org)^80^ accession numbers were processed through Database for Annotation, Visualization & Integrated Discovery (DAVID) bioinformatics resources v.6.7 (https://david.ncifcrf.gov/)^81,82^ and Kyoto Encyclopaedia of Genes and Genomes (KEGG) pathway maps database (http://www.genome.jp/kegg)^83,84^. For all enrichment analyses, a Benjamini–Hochberg corrected *p*-value ≤ 0.05 was considered strongly enriched.

### Statistics and reproducibility

All survival data were analysed by Kaplan–Meier Log-rank Test for overall survival and by the Student’s *t*-test for mean and maximum lifespan using Graph Pad Prism. Other comparisons were determined either by Student’s *t*-test or One-way ANOVA followed by post hoc *t*-test or Fisher’s LSD test, with *p* < 0.05 considered statistically significant.

### Reporting summary

Further information on research design is available in the Nature Portfolio Reporting Summary linked to this article.

### Supplementary information

Supplementary Information

Description of Supplementary Materials

Supplementary Data 1

Reporting Summary

## Data Availability

The mass spectrometry proteomics data have been deposited to the ProteomeXchange Consortium via the PRIDE^85^ partner repository with the dataset identifier PXD036857. The data that support the findings of this study are available in Supplementary Data 1. The uncropped and unedited blots/gels are shown in Supplementary Figs. 8–13, in the Supplementary Information.
