# Supplementary material for: The Drosophila ZNRF1/2 homologue, detour, interacts with HOPS complex and regulates autophagy
Source: Commun Biol. 2024 Feb 15;7:183. doi: 10.1038/s42003-024-05834-1 (PMC10869362; doi:10.1038/s42003-024-05834-1)
Supplement: Supplementary file 1 — Supplementary Information [file 42003_2024_5834_MOESM1_ESM.pdf]

SUPPLEMENTARY INFORMATION

The *Drosophila* ZNRF1/2 homologue, detour, interacts with HOPS complex and regulates autophagy

Shannon Nicolson, Jantina A Manning, Yoon Lim, Xin Jiang, Erica Kolze, Sonia Dayan, Ruchi Umargamwala, Tianqi Xu, Jarrod J Sandow, Andrew I Webb, Sharad Kumar, and Donna Denton

This Supplementary Information file contains:

Supplementary Figures 1 - 13

Supplementary Tables 1 - 2

Supplementary Figure 1

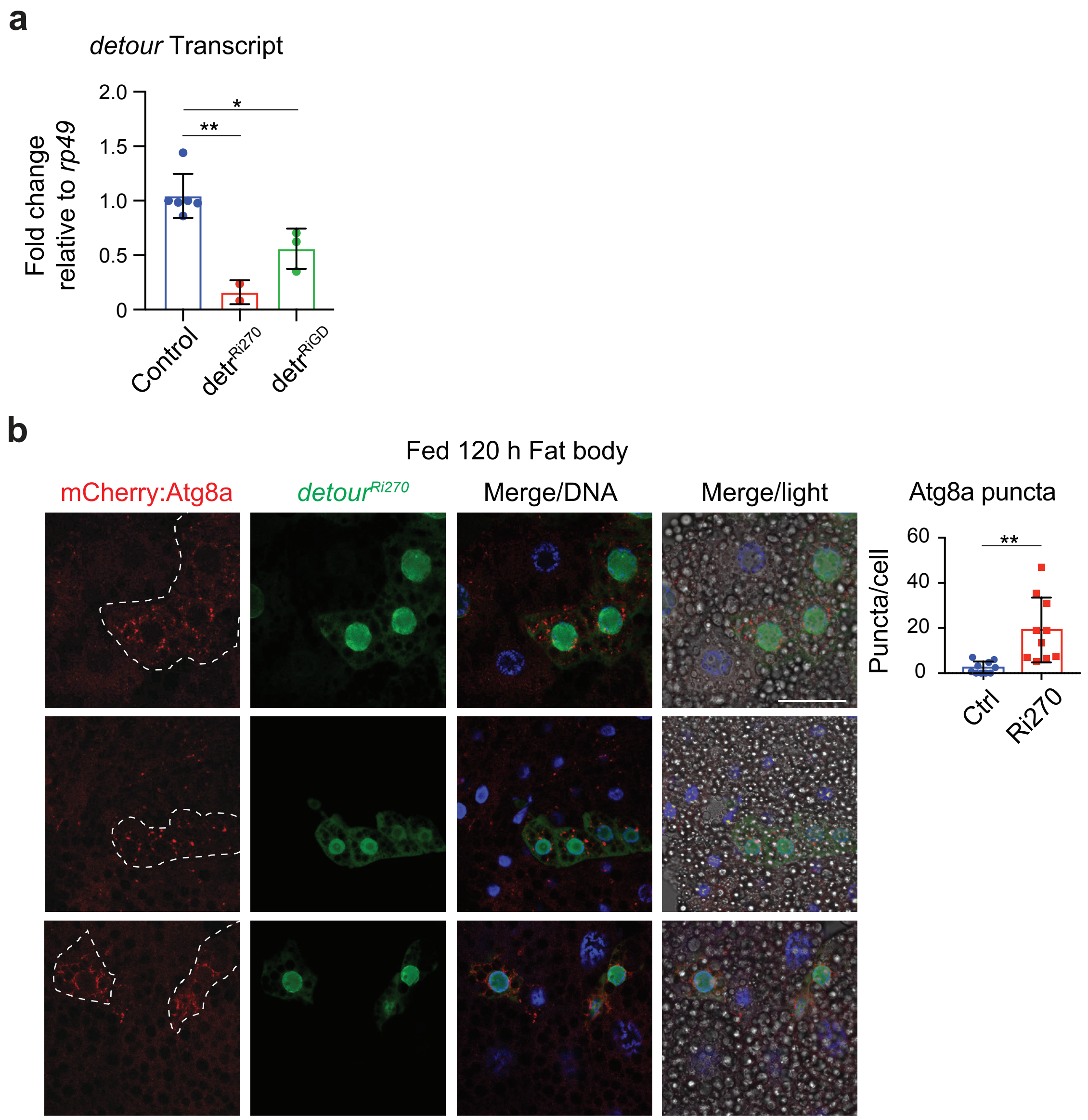

Supplementary Figure 1: detour ablation increases autophagic vesicles.

(a) Verification of *detour* knockdown in larval midgut from the third instar stage (-4 h RPF) by qRT-PCR. The levels of transcript of each knockdown line were normalised against the housekeeping gene *rp49* and are represented as fold change ± SD (*p<0.05, **p<0.01). Experiments were performed with at least 10 midguts per genotype.

(b) Fat body cells of *detour* knockdown (*hsFLP; pmCherry-Atg8a/+; Act>CD2>GAL4, UAS-nlsGFP/UAS-detour Ri270)* marked by GFP (green) have increased mCherry-Atg8a puncta (red, outlined) compared to the neighbouring control cells from feeding larvae fed at 120h AED. Three representative panels shown. DNA is stained by Hoechst (blue). Scale bar = 20 μm. Quantitation of puncta number represented as puncta/cell ± SD (**p=0.002).

Supplementary Figure 2

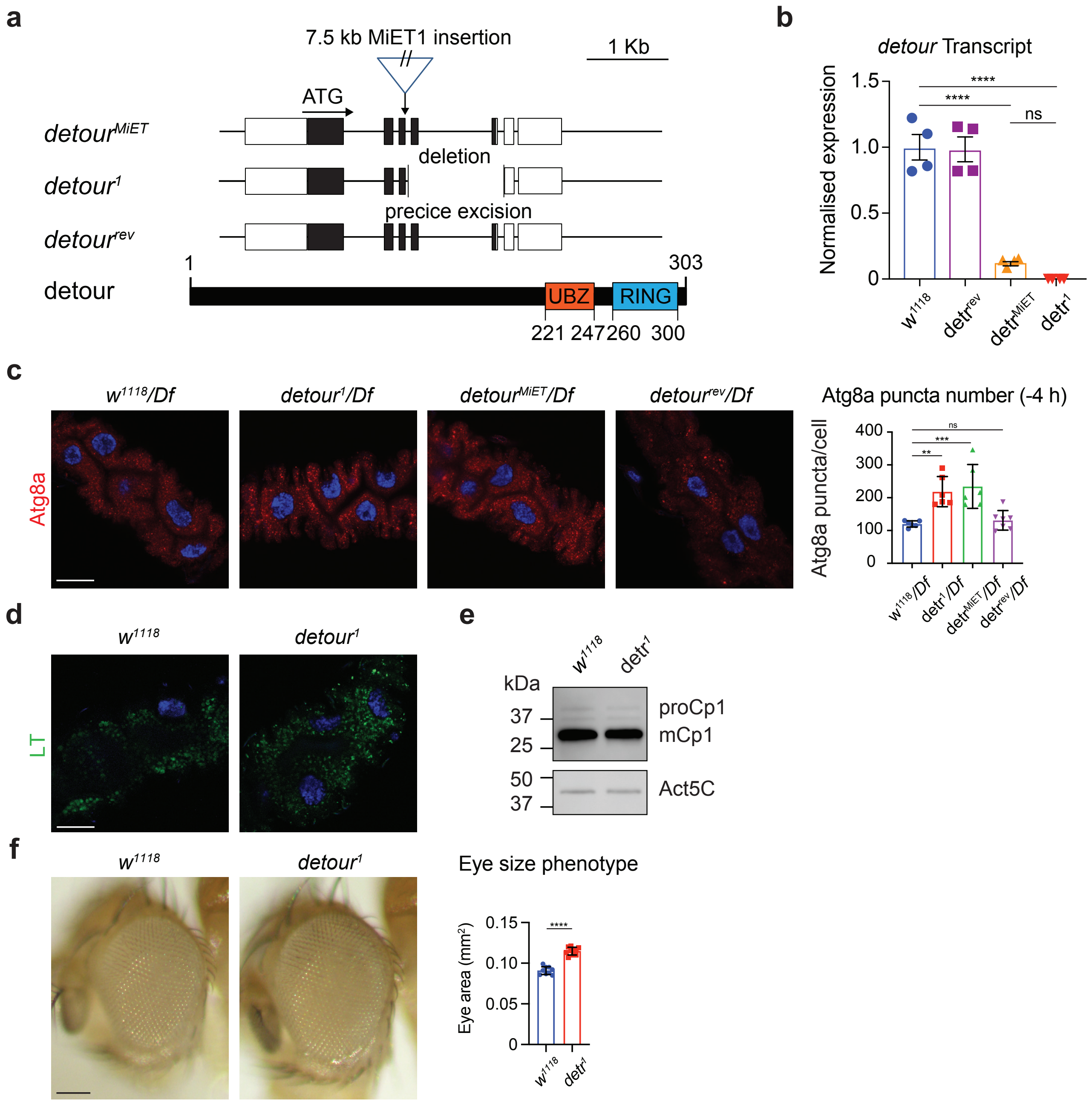

Supplementary Figure 2: *detour* mutants have increased autophagic vesicles.

(a) Schematic representation of detour alleles and protein. The *detour*^{MiET} contains a transgenic insertion of a Minos-based construct *Mi{ET1}* element. *CG144535*^{1} removes exons 4 and 5 of the *detour* gene without disrupting the adjacent *nonC* and *CG4557* genes. The precise excision allele, *detour*^{rev} contains a wild type ORF. The protein structure of detour showing the Ubiquitin-Binding Zinc finger (UBZ) and Really Interesting New Gene (RING) domains.

(b) Expression analysis of *detour* mutant lines in adult females by qRT-PCR. The *detour*^{rev} line had transcript levels similar to the control (w^{1118}) (ns p=0.9984) with *detour*^{MiET} and *CG144535*^{1} lines

showing a significant reduction compared with control (****$p<0.0001$). No significant difference was observed between *detour*^{MiET} and *detour* ^{1}. Experiments were performed four times with three female adult flies per genotype, error bars SEM.

(c) The Atg8a immunostaining (red) of midguts at -4 h RPF shows that *detour*^{1}*/Df* and *detour*^{MiET}*/Df* have similar levels of Atg8a puncta which are both higher compared to the controls *w*^{1118}*/Df* and *detour*^{rev}*/Df.* DNA is stained by Hoechst (blue). Scale bar = 20 µm. Quantitation of mCherry-Atg8a positive puncta represented as puncta/cell ± SD (**$p<0.01$, ***$p<0.001$).

(d) LysoTracker staining (green) of control and *detour* ^{1} midguts at -4 h RPF with nuclei stained by Hoechst (blue). Scale bar = 20 µm.

(e) Immunoblot analysis of Cathepsin L (Cp1) protein levels in lysates from control and *detour*^{1} adults with Act5C (Actin 5 C) as load control. The inactive proform of Cp1 (proCp1) is ~40 kDa and the mature form (mCp1) is ~35 kDa.

(f) The adult eye of *detour*^{1} is larger than *w*^{1118}. Scale bar = 100 µm. Quantitation of eye size represented as area ± SD (****$p<0.0001$).

Supplementary Figure 3

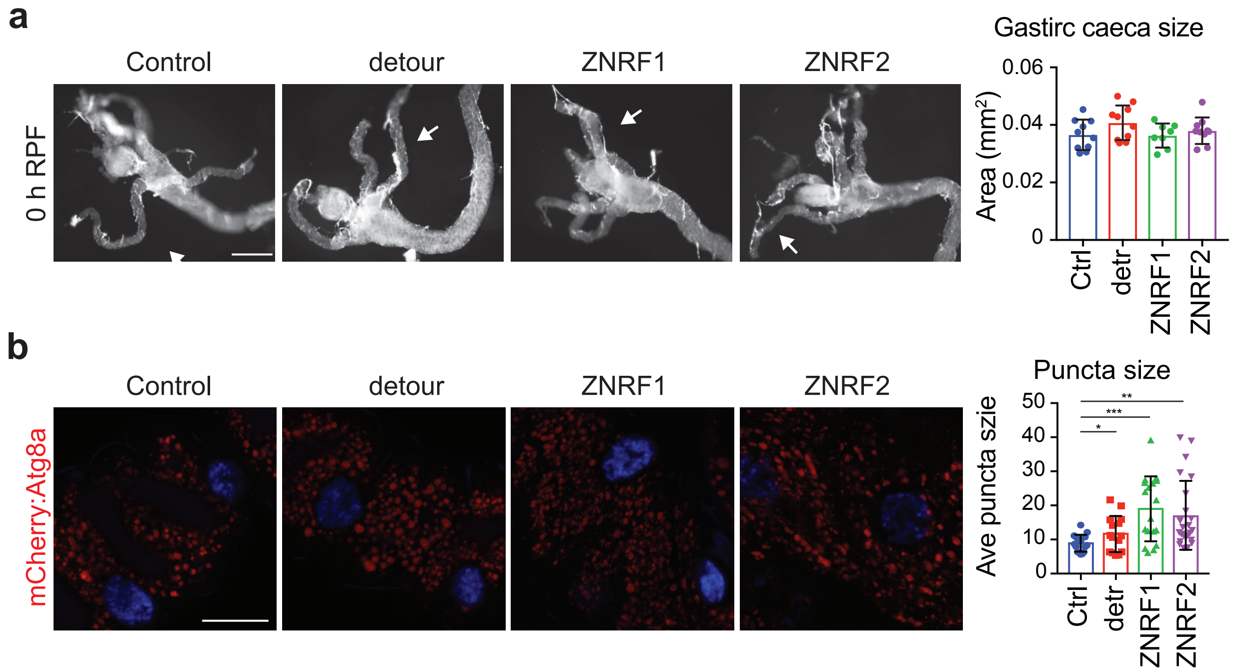

Supplementary Figure 3: Overexpression of detour, ZNRF1 or ZNRF2 increases autophagic vesicle size.

(a) Morphology from control (*w*[1118]), detour, ZNRF1 or ZNRF2 midguts at 0 h RPF show similar gastric caeca size (arrow). Scale bar = 200 µm. Quantification of gastric caeca size presented as average area ± SD.

(b) The overexpression of detour (*Mex-GAL4/+; pmCherry-Atg8a/UAS-detour:GFP*), ZNRF1 (*Mex-GAL4/UAS-ZNRF1; pmCherry-Atg8a/+*) or ZNRF2 (*Mex-GAL4/UAS-ZNRF2; pmCherry-Atg8a/+*) has increased mCherry-Atg8a (red) puncta size in the larval midguts at -4 h RPF compared to control (*Mex-GAL4/+; pmCherry-Atg8a/+*). DNA is stained with Hoechst (blue). Scale bar = 20 µm. Quantification of mCherry-Atg8a puncta size, measured in ImageJ. Data presented as average puncta ± SD, n=10 (*** p = 0.0001, **** p <0.0001).

Supplementary Figure 4

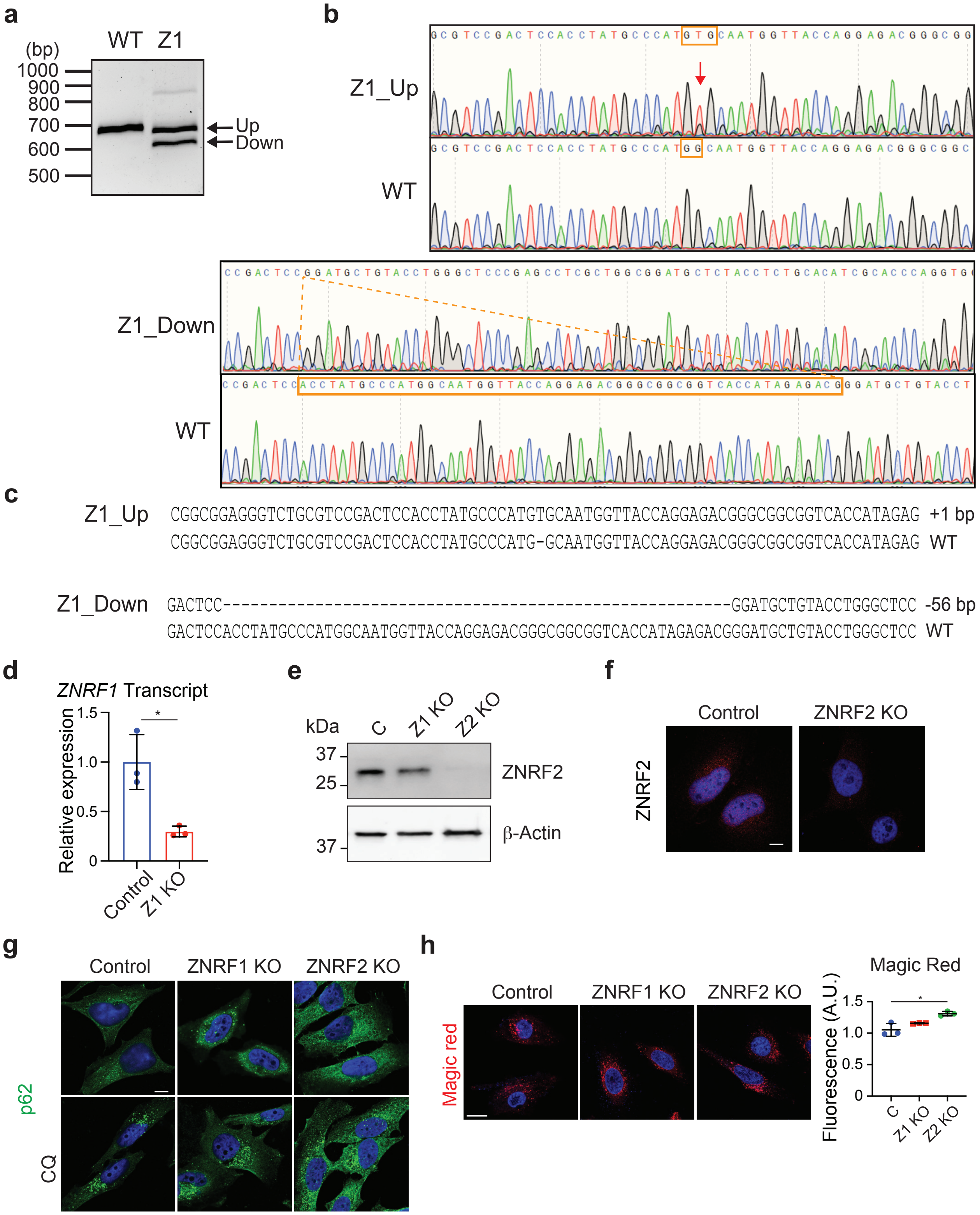

Supplementary Figure 4: Validation of *ZNRF1* and 2 KO HeLa cell line.

(a) A representative DNA agarose gel image from PCR of WT (W6) and ZNRF1 KO (B8) clones. "Up" and "Down" DNA fragments generated from B8 are indicated. DNA size marker (bp).

(b) DNA sequence chromatogram of purified PCR products shown in (a). The orange rectangle and red arrow indicate 1 bp insertion (B8_Up) and 56 bp deletion (B8_Down).

(c) DNA sequence alignment of WT and KO (B8_Up and B8_Down).

(d) Transcript levels of *ZNRF1* mRNA measured by RT-qPCR relative to *β-actin. ZNRF1* KO had significantly decreased *ZNRF1* transcript levels. Average ± SEM (* $p = 0.01$).

(e) Immunoblot analysis of ZNRF2 protein levels in whole cell lysates validates *ZNRF2* knockout in CRISPR/Cas9 HeLa cell line, β-Actin used as loading control.

(f) Immunostaining of control and *ZNRF2* KO HeLa cells with ZNRF2 antibody (red) merged with nuclei stained by Hoechst (blue). Scale bar = 10µm.

(g) Immunostaining of control, *ZNRF1* and *ZNRF2* KO HeLa cells with p62 antibody (green) merged with nuclei stained by Hoechst (blue) with or without 4 h of chloroquine (CQ) treatment. Scale bar = 10 µm.

(h) Live imaging of Magic Red stained (Red) control, *ZNRF1* and *ZNRF2* KO HeLa cells with nuclei stained by Hoechst (blue). Scale bar = 10µm. The fluorescence intensity of Magic Red-stained cells detected by plate reader. Average ± SD (* $p = 0.01$).

Supplementary Figure 5

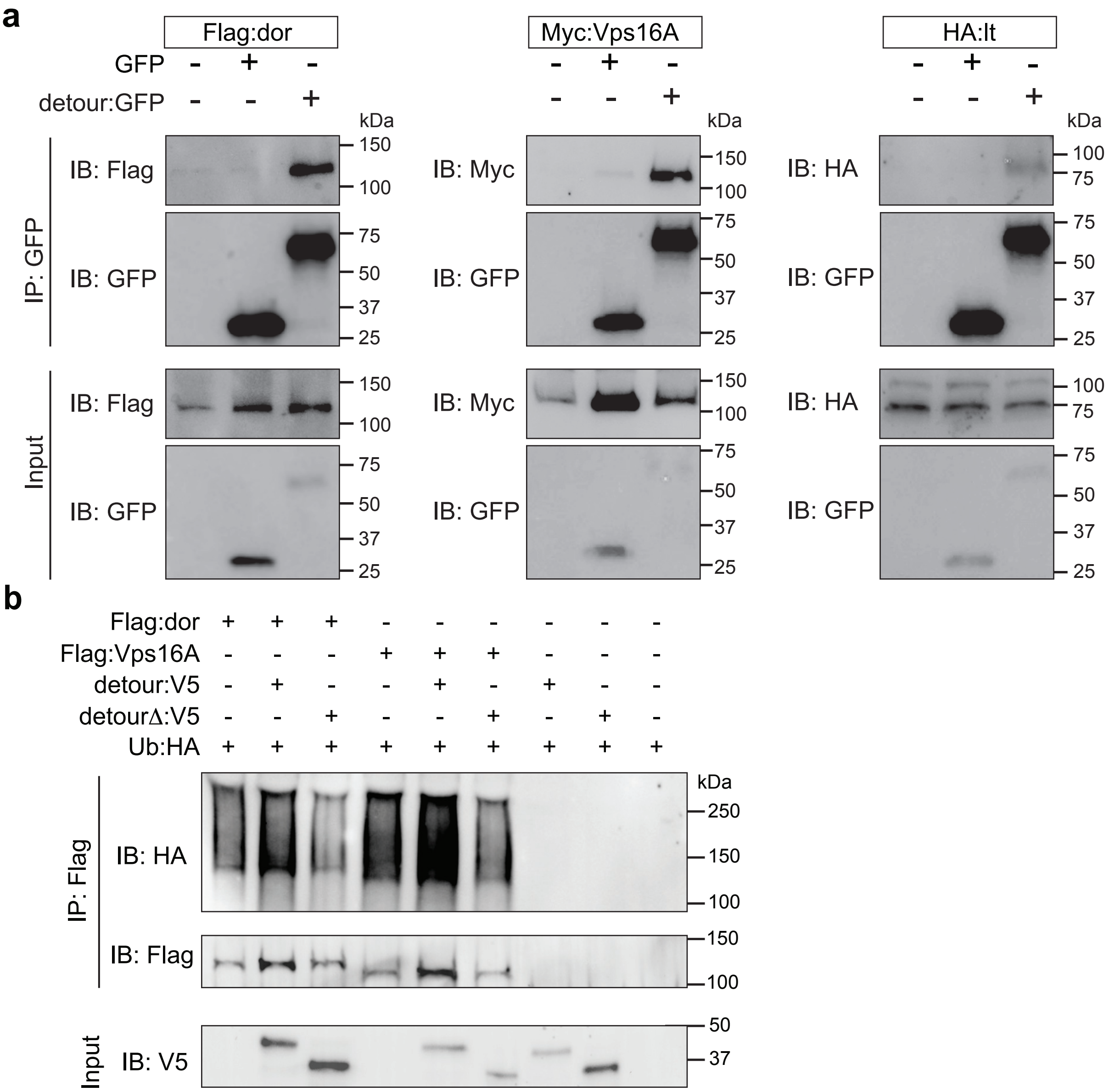

Supplementary Figure 5: detour interacts with the *Drosophila* HOPS complex.

(a) Lysates from SL2 cells co-transfected with GFP-tagged detour or GFP alone and Flag-tagged dor, Myc-tagged Vps16A or HA-tagged lt were subjected to immunoprecipitation (IP) with anti-GFP antibody. Proteins were separated by SDS-PAGE and immunoblotted (IB) with anti-GFP antibody and anti-Flag, anti-Myc or anti-HA antibody. Input controls were 5% of each protein lysate.

(b) Lysates from SL2 cells co-transfected with V5-tagged detour (detour:V5) or V5-tagged detour RING deletion (detourΔ:V5) and Flag-tagged dor or Flag-tagged Vps16A with HA-tagged ubiquitin were subjected to IP with anti-Flag antibody and IB with anti-Flag, anti-HA and anti-V5 antibody.

Supplementary Figure 6

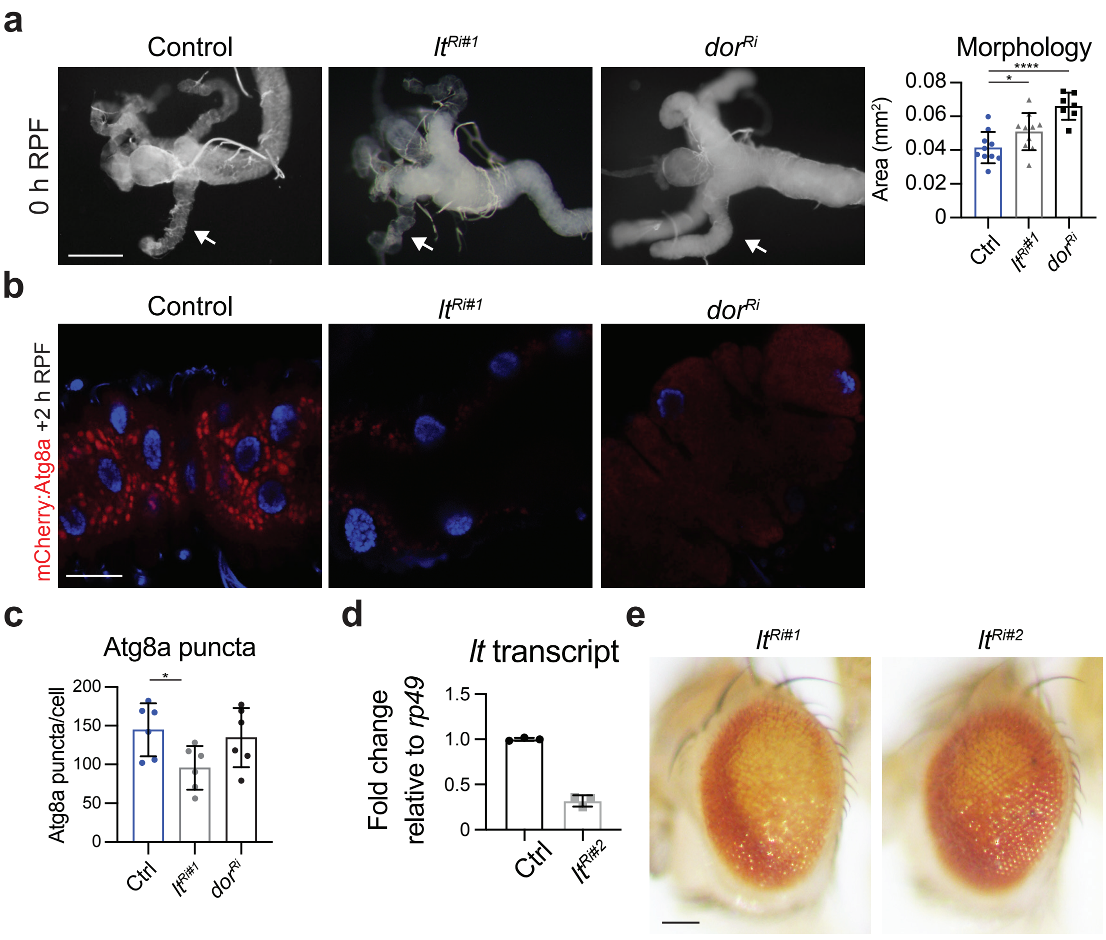

Supplementary Figure 6: *detour* interacts with *light*.

(a) Morphology from control, *dor* knockdown (*dor*^{Ri}) and *lt* knockdown (*lt*$^{Ri\#1}$) midguts at 0 h RPF show increased gastric caeca size (arrow). Scale bar = 200 µm. Quantification of gastric caeca size presented as average area ± SD. (*p<0.05; ****p<0.0001).

(b) The knockdown of *lt* (*Mex-GAL4/UAS-ltRi*#1*; pmCherry-Atg8a/*+) and *dor* (*Mex-GAL4/+; pmCherry-Atg8a/UAS-dorRi*) have decreased mCherry-Atg8a (red) puncta in the larval midguts at +2 h RPF compared to controls (*Mex-GAL4/+; pmCherry-Atg8a/*+). DNA is stained with Hoechst (blue). Scale bar = 20 µm.

(c) Quantification of Atg8a puncta number from Figure 7c, measured in ImageJ. Data presented as average puncta ± SD (*p<0.05).

(d) Verification of *lt* knockdown line #2 ($lt^{Ri\#2}$) in larval midgut from the third instar stage (-4 h RPF) by qRT-PCR. The level of *lt* transcript was normalised against the housekeeping gene *rp49* and represented as fold change ± SE. Experiments were performed with at least 10 midguts per genotype. **(e)** The knockdown of $lt^{Ri\#1}$ and $lt^{Ri\#2}$ in the developing eye results in a pale eye colour phenotype, with loss of pigmentation. The pale eye phenotype is stronger in the $lt^{Ri\#2}$ line consistent with a greater level of knockdown from Line #2 compared to Line #1.

Supplementary Figure 7

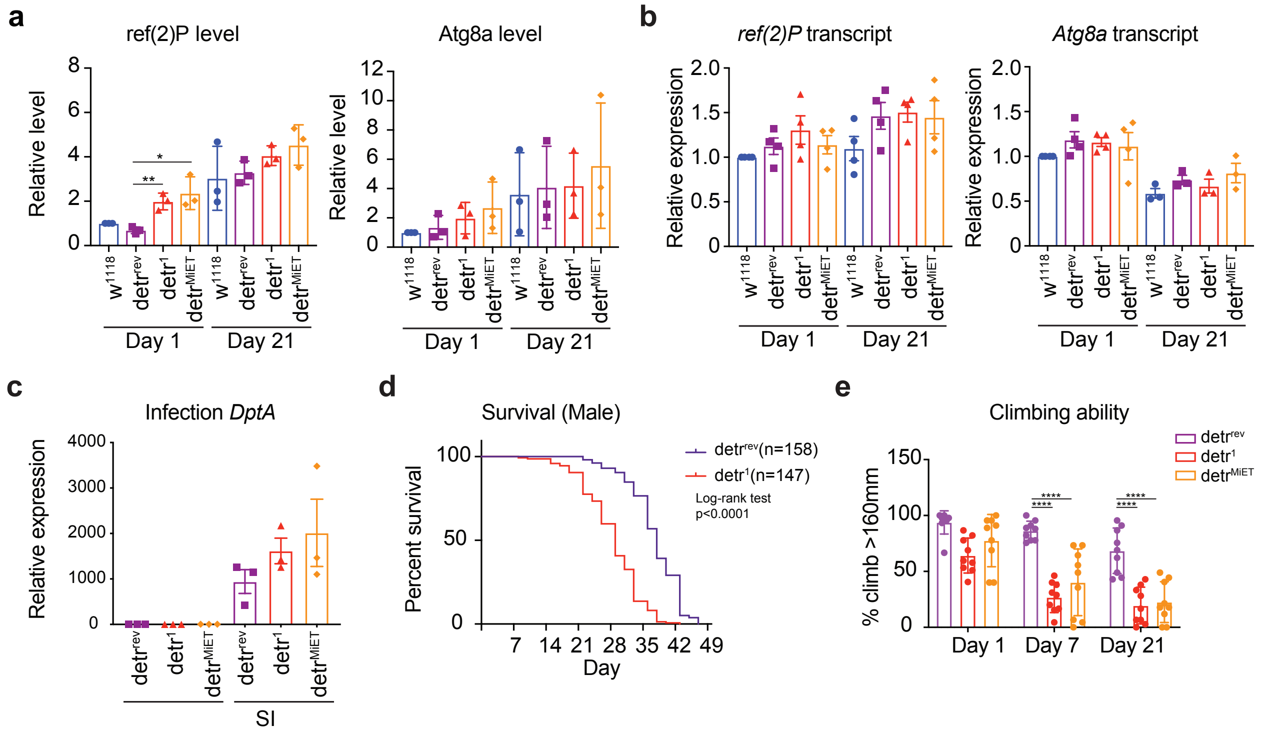

Supplementary Figure 7: detour regulates autophagy and is required for healthy ageing.

(a) The combined quantitation of immunoblots showing ref(2)P and Atg8a protein levels in young (day1, as shown in Fig. 9b) and aged (day 21) *detour* mutant and control adults. Data presented as mean relative intensity ± SD (n = 3 individual repeat experiments; *p<0.05, **p< 0.01).

(b) Transcript level of *ref*(2)p and *Atg8a* was examined by qRT-PCR from larval midgut (-4 h RPF). The levels of transcript were normalized against the housekeeping gene *rp49* and are represented as fold change ± SEM. Experiments were performed with at least 10 midguts per genotype.

(c) The transcript levels of *DptA* from control and *detour* mutant young (0-3 day) adults following septic injury was measured by qRT-PCR. The levels of transcript were normalised against the housekeeping gene *rp49* from samples containing 3/5 adults per group.

(d) Survival assays for male isogenic control (*$detour^{rev}$*) and *$detour^{1}$* adults. Kaplan-Meier survival assay presented as percentage of surviving population per time point (Log-rank p<0.0001; Gehan-Breslow-Wilcoxon p<0.0001).

(e) The percent of male flies unable to climb above 160mm (Climbing ability) in a cylinder after 25 seconds was determined every week. Data presented as mean ± SD, n ≥ 45 flies per experiment. (*p< 0.05; **** p < 0.0001).

Supplementary Figure 8

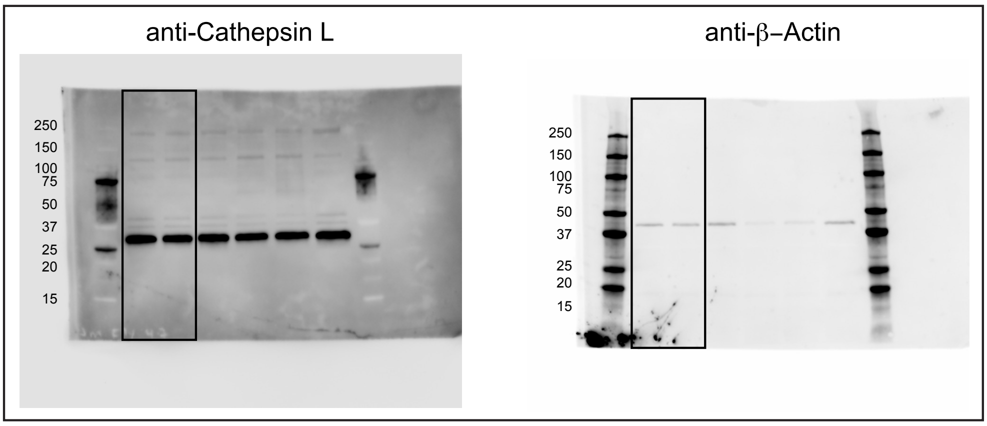

Supplementary Figure 8: Scanned images of western blots with markers.

Western blot images showing representative lanes (outlined) and markers (kDa) for Supplementary Figure 2e.

Supplementary Figure 9

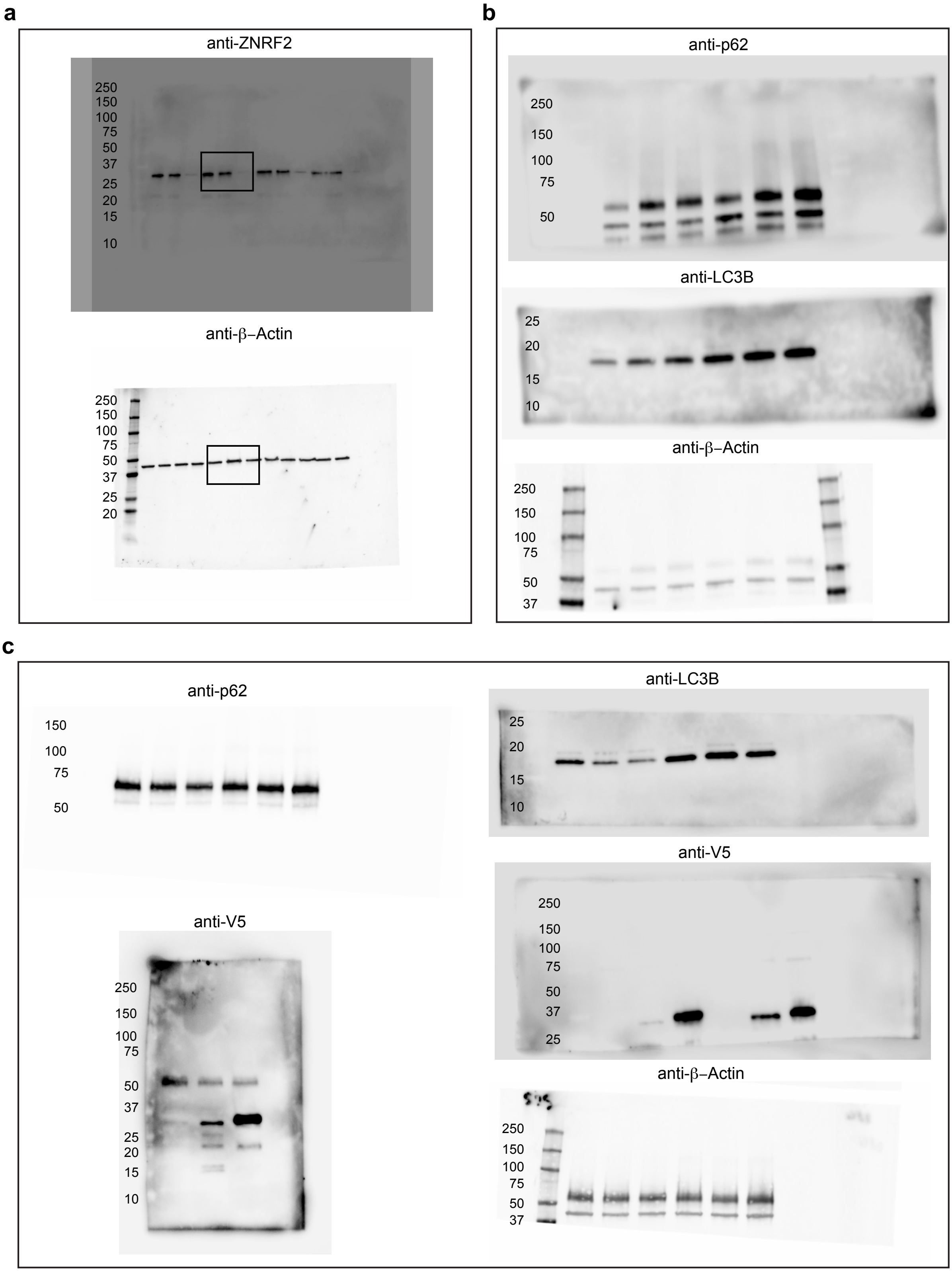

Supplementary Figure 9: Scanned images of western blots with markers.

(a) Western blot images showing lanes (outlined) and markers (kDa) for Supplementary Figure 4e.

(b) Western blot images showing markers (kDa) for Figure 4a.

(c) Western blot images showing markers (kDa) for Figure 4f.

Supplementary Figure 10

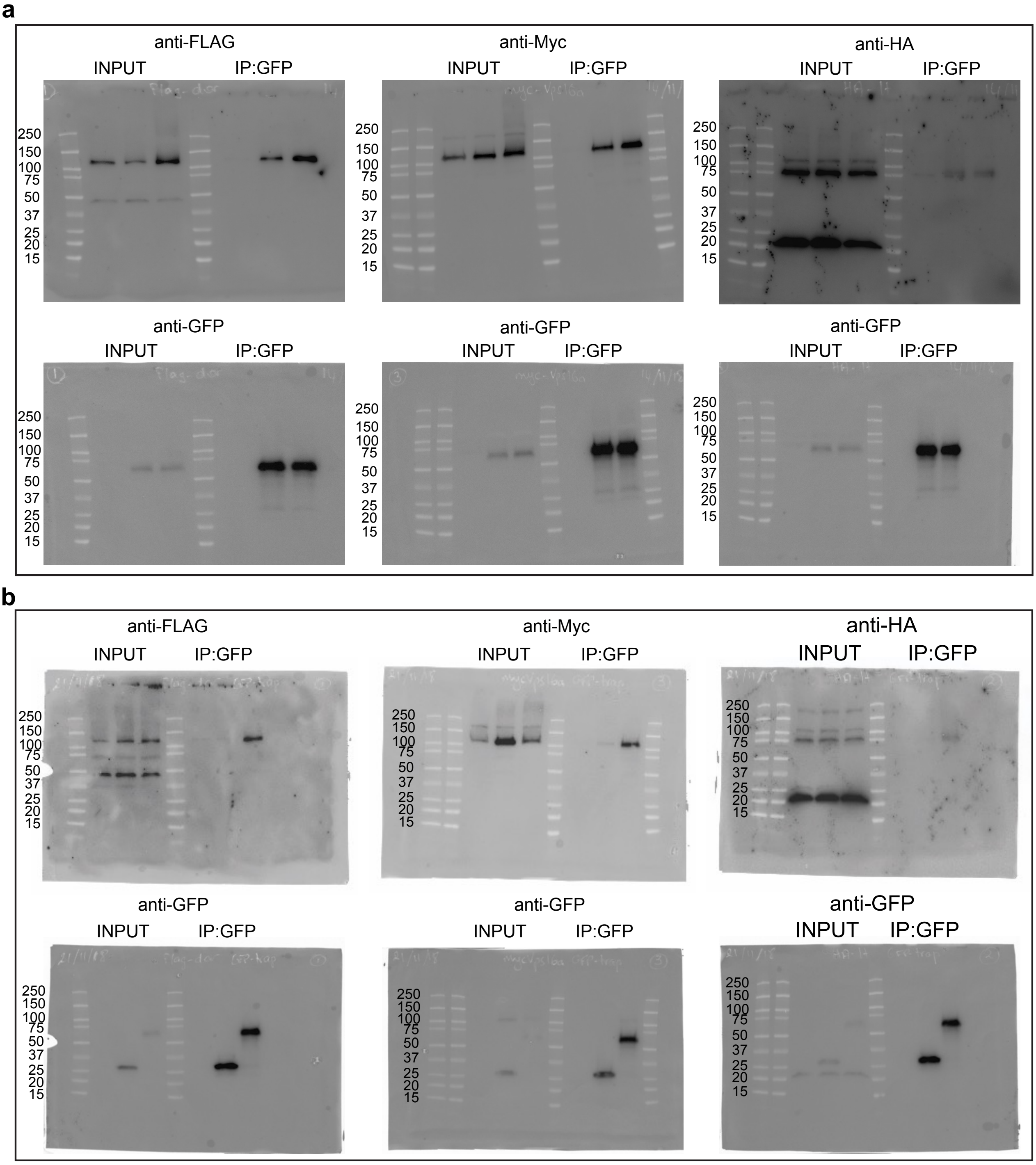

Supplementary Figure 10: Scanned images of western blots with markers.

(a) Western blot images showing markers (kDa) for Figure 6a.

(b) Western blot images showing markers (kDa) for Supplementary Figure 5a.

Supplementary Figure 11

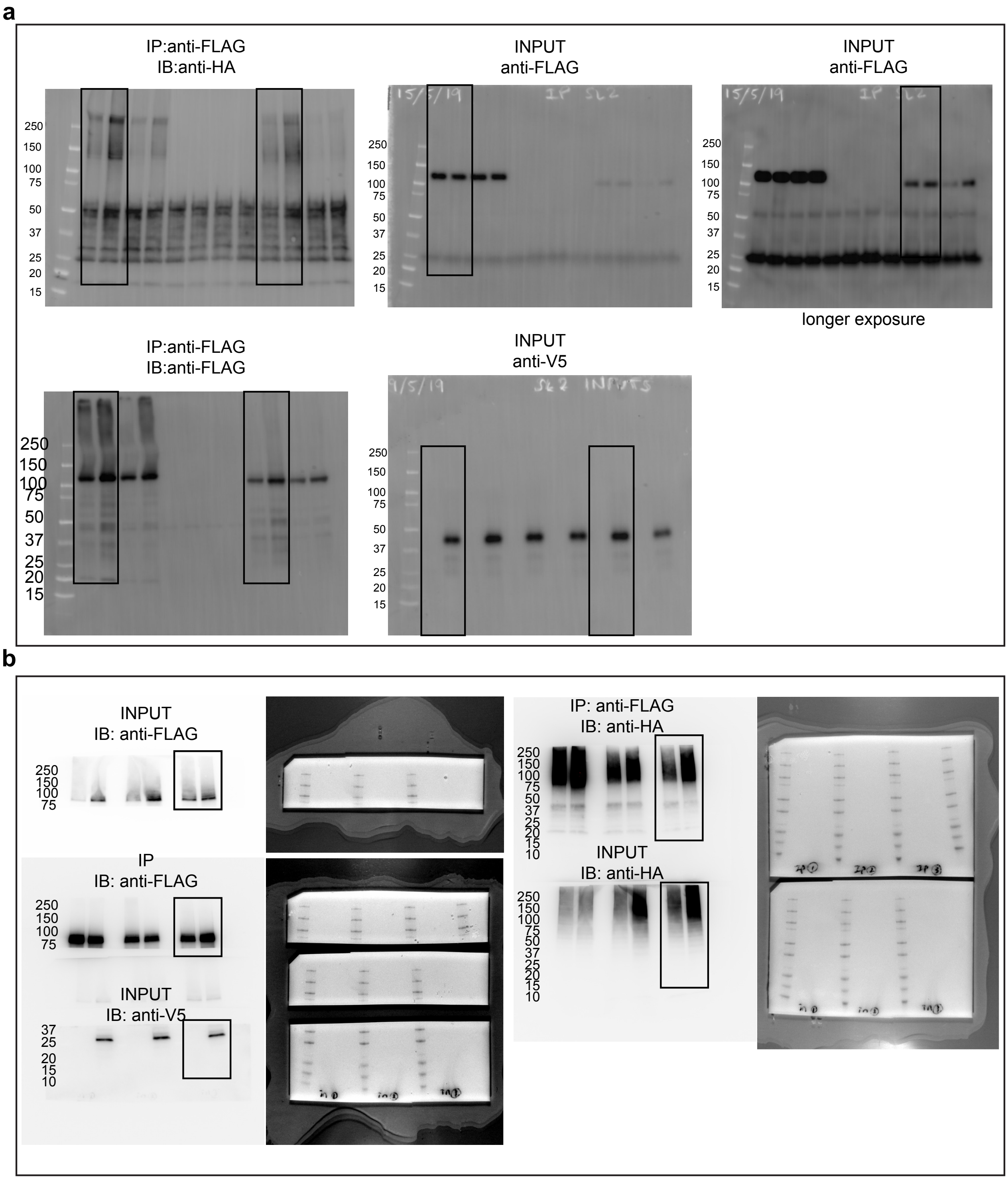

Supplementary Figure 11: Scanned images of western blots with markers.

(a) Western blot images showing lanes (outlined) and markers (kDa) for Figure 6b, right hand side and middle panels.

(b) Western blot images showing lanes (outlined) and markers (kDa) for Figure 6b, left hand side panel.

Supplementary Figure 12

a

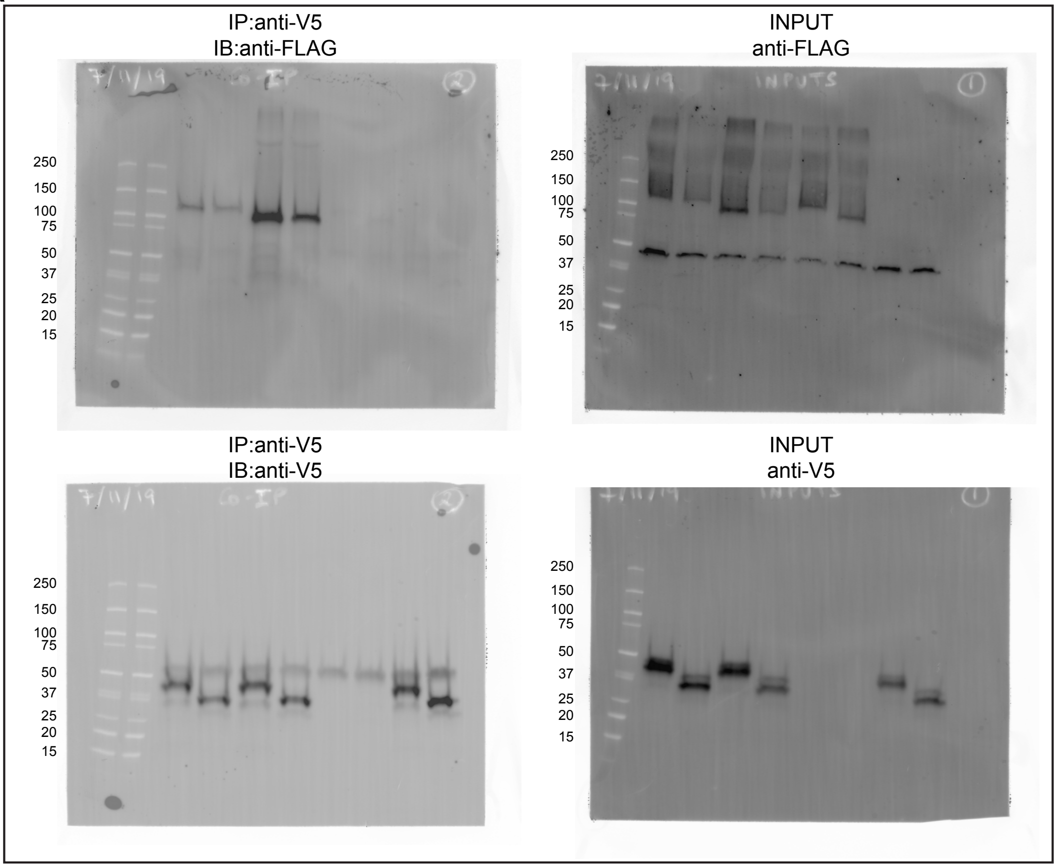

b

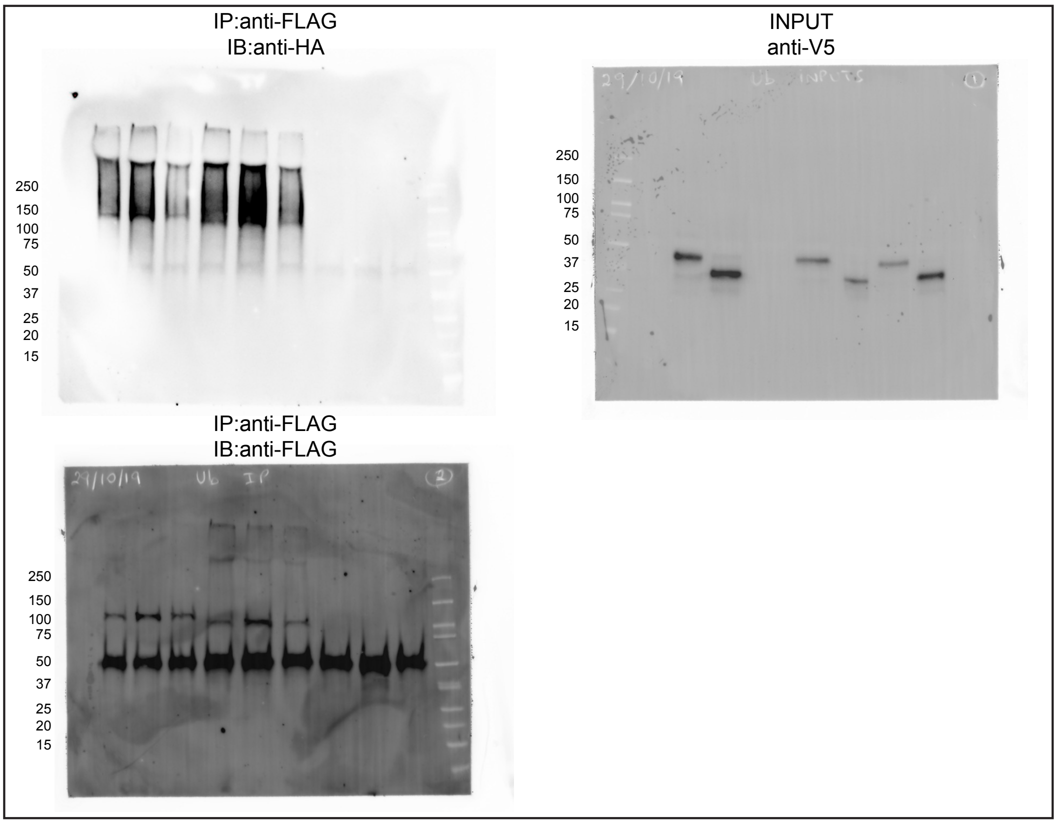

Supplementary Figure 12: Scanned images of western blots with markers.

(a) Western blot images showing markers (kDa) for Figure 6c.

(b) Western blot images showing markers (kDa) for Supplementary Figure 5b.

Supplementary Figure 13

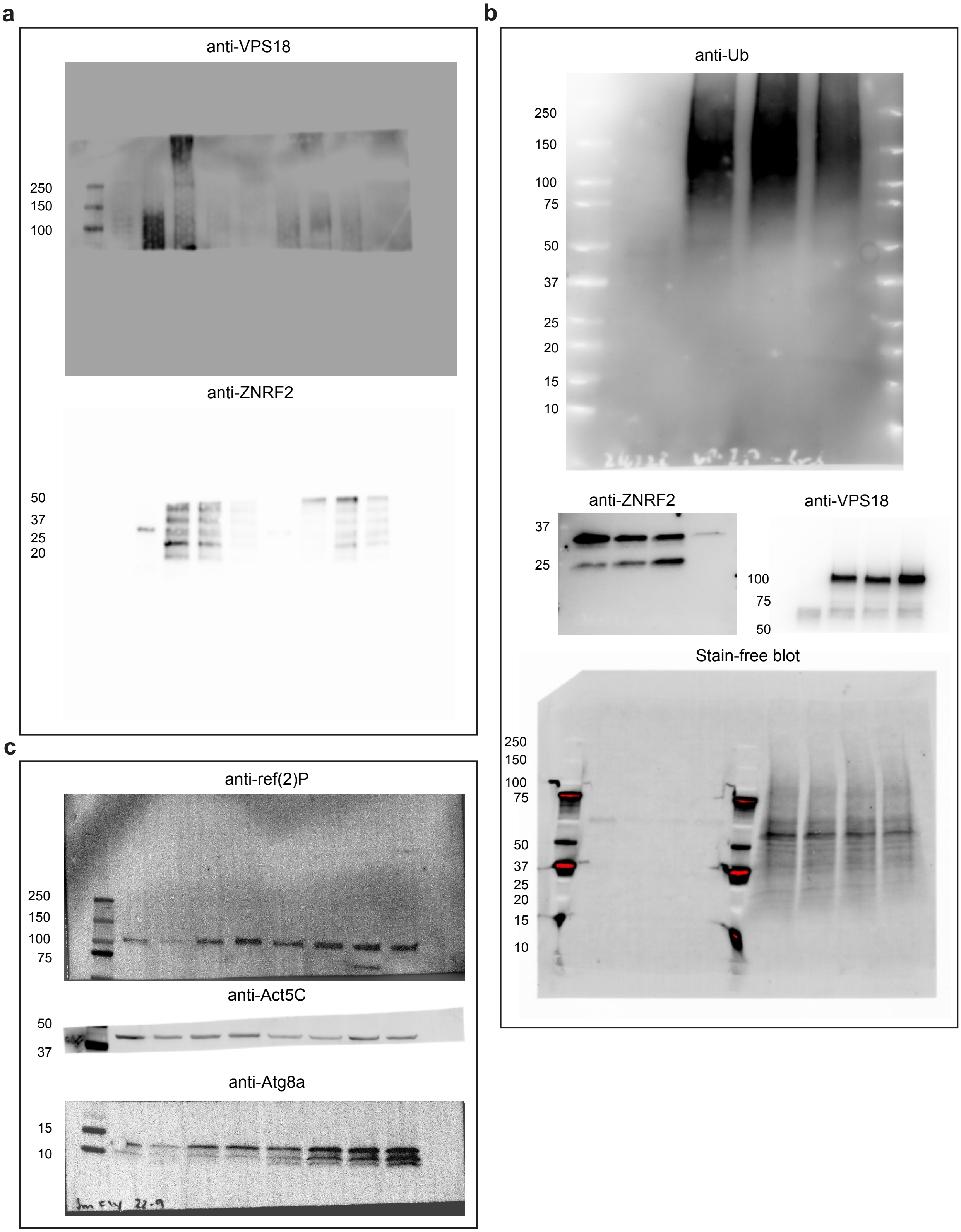

Supplementary Figure 13: Scanned images of western blots with markers.

(a) Western blot images showing markers (kDa) for Figure 8c.

(b) Western blot images showing markers (kDa) for Supplementary Figure 8d.

(c) Western blot images showing markers (kDa) for Figure 9.

Supplementary Table 1

Supplementary Table 1: gBlock® sequence used for Gateway® cloning

Insert	Sequence
**detour**	CACCATGGGTCAAAAGGCGAGCACTCCGGCGACCAGTGGCCAACAGAGCCCA<br>CGCTCCAGGACATTCTCGAGCAGTTCAACAGGAGCCGCCGATTCTGCGCTGCA<br>GCAGCAGCAACACCAGCACTCGAATCCCGGCCAGAATCGTGGCGGTAACGGC<br>GGCGGCGGTGGCGGCAATGATACCACCGGTCAGGGTTTCAATCTGCTGCGCAC<br>TTTGCCCGGCCTGCAGGTGTACCACAACCAAAACTCCACGTCCATCGATCGCC<br>AGCGGGCGCGGAGTCTCAGCTCCGTGCCGGATATACAGCAGCAGGCGCAGCA<br>GCAGCAGCAACAGGGATCGATCACCTCGTCCGGCAGCAGTCCGCATTCGCATC<br>CGCATGCCGCGCACGGCCATCCGGCCGTCGGTGTCACCGTATCCGTGTCCGGT<br>AATGCGAATGCCAATAGCAACAGCAACTTCACAGCCGCCGGGAACAACAATG<br>GATCCGCCATGCAGTCCTACATGCAGCAGAGACGCACTCTCGGCGATTCCATA<br>AGGGACATGTCAATGACCGGCGGCAGCATCGCCGAGAGCATCGCCCTGGCCG<br>CTTCCGCCACGTCCGCCAATGGGATCGGTCGTGTCTACACGGCCACCTCGCTTC<br>CATCGCACATTTGGTCCTTCAATGGTATCAAGTGTCCCGTGTGCAATAAGTTTG<br>TGTTGCCAGACGACATCGAATGCCATTTAGTCATGTGCCTAACGAAACCACGA<br>CTCTCATATAACGAGGACGTACTCTCGGACGCCAAGGGCGAGTGCGTCATTTG<br>CCTGGAGGATCTGAGTCCGGGGGACACCATCGCCCGGCTGCCATGCCTCTGCA<br>TATATCACAAAGGATGCATCGACCGTTGGTTTGAGGTGAATCGCTCTTGCCCT<br>GAGCATCCTGGAGATAAGAAA
**lt**	CACCATGGCTAAAGCGTTGCCGCTCATCAGTTGTGACTCTTGGGCCGATAGCA<br>TAAATGAGGAAGATGTGGAGCCCAAATTTAAGTATCACCGTCTCGCAAATGAT<br>TTAAAATACATGCTCAACGCTGATGTCATCACTTGTAGCGCTGTACACTTAAA<br>GTTTCTTATATTTGGAACTTTCCGAGGACGTGTTTGTATATTTGACCATCAAGG<br>AAACTCAGTCTACTCAAATCTTAGCGCTAGCGAACGTCACACTCACCAAGTGG<br>CAGTAAACAATATAGATGTAGATCATAAGGGCGAGTATGTGGCCACATGCTCC<br>GACGACGGAAAAGTTAACATAACGGGATTGTTTAGCTCTGATAACAATCACAG<br>TCTCAGCTTTGGCAAATTTATAAAGGTCGTATCACTGGAGCCCGATTCCAAAG<br>CTCACATTAAAAGATTTGTAGTGGGCGACGACAAACTTATCTTGTACGAGCGA<br>AACTTGCTAAAGAAGCTTAAACCAGTTGAACTGTGCTCAGTCGAAGGCAGTGT<br>TTTGTCAATCTGCTGGCATGGCAACTTTATTGCCTGGGCAAGTCACATAGGAGT<br>TCGAGTCTACGATTTAAACGAAAGATGCTCACTTGGATTAATAAAGTGGGAGG<br>TTCCTCCTCAGGAGCGATTAGAAAATTTTCGATGCCACCTTCGTTGGTCCAACA<br>AGCATACATTATTAATTGGCTGGGTAGACACTATTCGAGTTTGCGTAATTCGA<br>AAGAGAAATTCAATCGAAGCTTCAACTGGCAATCTGCCTGTCTACATTGTGGA<br>TCCAATCTCGACATTTCAAACAACTTTCTACGTATGTGGGCTTGCACCATTATC<br>TGCAAAGCAATTGGTTGTTCTTGGCTTTCGCAAGGAAAAAAGCTCTTGTTTTAA<br>AGCCCTACGACCTGTATTATGCGTTATTGAATACAAAATGAATAATAGCGAGG<br>AAATCTGCACAGACAGTTTAACTTTACGCGGCTTTGAGGAGTACACCGTGAAT<br>GACTATAGTCTGGGTGGCATAATCGAGGAGAATCGCTTTTATATTGTGGCCCC<br>AAAGGATATTGTGGTAGCTAGTCTTATTGAAACAGATGATCGTATAGAATGGT<br>TGATTAAACACAGTAAATTTGAGGAGGCAATGGAACTTATATCTGCGAATGGA<br>GGTAACGTTCCCGTACTTTCCGTTGCTAAACTTTATATCAACCATTTACTGGCG<br>CTTAAGAAGTATGATGACGCAGCAAAACTTTGCCTCCGCATGCTAGGCAATGA<br>CAAAGTCCTTTGGGAAGAGGAAGTTTTTAAGTTTGTAAAGTGTCAGCAGCTAC<br>GATCAGTTAGCGCCTATCTGCCGACTTCAGACGAATGCAAACTTGATCCCCAT<br>GTATACGAAATGGTTTTATACGAGTTTCTCAAATTTGATGTATGTGGTTTCCTG<br>AATCTTATCAAGGAATGGCCATCTCATCTTTATGACGGCCTGGCTGTTATTAAC<br>GCTATTCACGATAACTTTCGAAAGCACTATGCCAATCAGTTGCTGGAATCATT<br>GGCTCTTTTGTATTCCTATCAAGGCGACTTCGAAAGTGCTCTTCGCATGTATTT<br>AAAGTTGCAGAACAAGGATGTCTTCCAATTAATTCGACGGTATGAGCTTTACG<br>ATGTCATTTCAAAACTGATTATTCCACTTATTCAATTGGATCGTGACTGCGCCT<br>TTGAAATACTACTTGACAAGAAAAAAAATAAAGACTGAGATAGTTGTCCACCAG<br>CTAGAGCACAATCAAGAGTACCTGTACTGGTACTTAGATTCTTACTTAAAAA

	GGATCCCAGTAATGTGTTTCAAAAAAAACTTATCTCCTTATATGCCATTTTCGA<br>TCGAAACAAACTATTGCCCTTTCTAAAGCGTTCAAAGGACTATGACATTCAGG<br>AGGCGTTGGTCATATGCAAACAGGAAAACTTTTATCCAGAAATAGTCTACCTG<br>CTCGGGTGTATGGGTGGCGTAGAAGCGTCCGAAGCACTTAATATTATTATTCA<br>TCGCATCAGAGACATAGAAATGGCCATAGAGTTTTGCAAGGAGCACGATGAC<br>AGCGATCTTTGGAATGCACTTATAAATGAATTTAGCAAGCACCCTGAAATTGT<br>GACCAAAGTTTTGGACGGCATAGTTGACTACTTTAGCCCAGCGGTGGTTGTTG<br>GAAAGATCAAAATGGGACAGAACATTCCAAATCTTCGACAATCACTTATAAAA<br>ATGCTGCGCCACTACAATCTTCAAGGTGAAATTTTATCCTCCGCCCAACAAATT<br>CAACTTAATGACTATTTTGAAATTCACTCGGAGATAGTCACCACGCAAAGACG<br>AGGTCAGCAGGTTTCCTACGAACAACTATGCTCATTGTGCCACCGTCCAGTAC<br>TGATGGCAGGCACACACTTGTATTGTATAATCAGGTTAGAGTGTGGTCATGTA<br>TACCATAAACCGTGCATACAAGGTGAATTGCTTAAAAATTGCAACGAGTGTAA<br>CCTTTGGAAGTTAACCGTGGGGAAATAG
**ZNRF2**	CACCATGGGCGCCAAACAGAGCGGCCCGGCAGCAGCTAACGGCCGCACGCGC<br>GCGTACTCGGGCTCGGATCTACCTTCCAGTAGCAGCGGAGGCGCCAATGGGAC<br>TGCTGGCGGTGGAGGCGGTGCTCGTGCTGCAGCTGCCGGAAGATTCCCAGCTC<br>AGGTGCCAAGCGCTCACCAGCCAAGCGCCTCAGGCGGTGCTGCCGCAGCTGCG<br>GCAGCTCCAGCGGCTCCAGCAGCTCCACGAAGCCGCTCCCTCGGAGGTGCTGT<br>GGGTAGCGTGGCATCGGGTGCTCGAGCAGCTCAGTCCCCCTTCAGCATCCCGA<br>ACAGCAGCAGCGGTCCGTACGGATCGCAGGACTCGGTGCACAGCAGCCCTGA<br>GGACGGAGGTGGCGGACGGGACCGTCCAGTGGGAGGGAGCCCAGGTGGTCCA<br>CGACTGGTGATCGGCTCCTTACCAGCTCACCTCTCGCCGCACATGTTTGGAGG<br>ATTTAAGTGCCCTGTATGCTCAAAATTTGTATCCTCAGATGAAATGGATTTGCA<br>TCTTGTAATGTGTTTAACAAAGCCACGAATAACCTATAATGAGGATGTACTGA<br>GTAAAGATGCTGGGGAATGTGCAATATGCCTTGAAGAATTGCAGCAGGGAGA<br>TACTATAGCACGACTGCCTTGTCTATGCATATATCATAAAGGCTGCATAGATG<br>AATGGTTTGAAGTAAATAGATCTTGCCCTGAGCACCCTTCAGAT
**V5**	CACCGGTAAGCCTATCCCTAACCCTCTCCTCGGTCTCGATTCTACG

Supplementary Table 2

Supplementary Table 2: Primers used for qRT-PCR.

**Target**	**Forward primer (5’-3’)**	**Reverse primer (5’-3’)**
detour	TGTGCCTAACGAAACCACGA	CTCAAACCAACGGTCGATGC
rp49	CCAGTCGGATCGATATGCTAA	ACGTTGTGCACCAGGAACTT
Atg8a	CATCGGTGATTTGGACAAGA	TGCCGTAAACATTCTCATCG
ref(2)p	AAACCACCACCGAAACAGAG	CCTCCTCATTCATGTGCTGA
AttA	GATGGACGTGCTAATCTCTG	GGCTTAGCCGAAATGATGAG
DptA	AGTTCACCATTGCCGTCGCC	GTAGGTGTAGGTGCTTCCCA
Dro	TCCACCACTCCAAGCACAATG	ACACATCTTTAGGCGGGCAG
Human		
ZNRF1	CTTGCTGACTCCTCTCAAAGGGAC	ATCTGGAAGTCAGTGCAGAAGG
β-actin	GATCATTGCTCCTCCTGAGC	AGTCCGCCTAGAAGCACTTG
