# Supplementary material for: The Drosophila ZNRF1/2 homologue, detour, interacts with HOPS complex and regulates autophagy
Source: Commun Biol. 2024 Feb 15;7:183. doi: 10.1038/s42003-024-05834-1 (PMC10869362; doi:10.1038/s42003-024-05834-1)
Supplement: Supplementary file 4 — Reporting Summary [file 42003_2024_5834_MOESM4_ESM.pdf]

nature portfolio

Corresponding author(s): Donna Denton

Last updated by author(s): Dec 22, 2023

Reporting Summary

Nature Portfolio wishes to improve the reproducibility of the work that we publish. This form provides structure for consistency and transparency in reporting. For further information on Nature Portfolio policies, see our Editorial Policies and the Editorial Policy Checklist.

Statistics

For all statistical analyses, confirm that the following items are present in the figure legend, table legend, main text, or Methods section.

n/a	Confirmed	
☐	☒	The exact sample size (*n*) for each experimental group/condition, given as a discrete number and unit of measurement
☐	☒	A statement on whether measurements were taken from distinct samples or whether the same sample was measured repeatedly
☐	☒	The statistical test(s) used AND whether they are one- or two-sided<br>*Only common tests should be described solely by name; describe more complex techniques in the Methods section.*
☒	☐	A description of all covariates tested
☒	☐	A description of any assumptions or corrections, such as tests of normality and adjustment for multiple comparisons
☐	☒	A full description of the statistical parameters including central tendency (e.g. means) or other basic estimates (e.g. regression coefficient) AND variation (e.g. standard deviation) or associated estimates of uncertainty (e.g. confidence intervals)
☒	☐	For null hypothesis testing, the test statistic (e.g. *F*, *t*, *r*) with confidence intervals, effect sizes, degrees of freedom and *P* value noted<br>*Give P values as exact values whenever suitable.*
☒	☐	For Bayesian analysis, information on the choice of priors and Markov chain Monte Carlo settings
☒	☐	For hierarchical and complex designs, identification of the appropriate level for tests and full reporting of outcomes
☒	☐	Estimates of effect sizes (e.g. Cohen's *d*, Pearson's *r*), indicating how they were calculated

Our web collection on statistics for biologists contains articles on many of the points above.

Software and code

Policy information about availability of computer code

Data collection	Zeiss ZEN software for microscopy image acquisition (ZEN 3.2 blue edition).<br>Rotor-Gene Q<br>ChemiDoc MP (BioRad)<br>SparkControl magellan (TECAN)
Data analysis	Fiji open source image processing software (vl.53t)<br>Microsoft Excel and GraphPad for statistical analysis<br>Image Lab (BioRad)<br>MaxQuant software version 1.5.8

For manuscripts utilizing custom algorithms or software that are central to the research but not yet described in published literature, software must be made available to editors and reviewers. We strongly encourage code deposition in a community repository (e.g. GitHub). See the Nature Portfolio guidelines for submitting code & software for further information.

Data

Policy information about availability of data

All manuscripts must include a data availability statement. This statement should provide the following information, where applicable:

- Accession codes, unique identifiers, or web links for publicly available datasets
- A description of any restrictions on data availability
- For clinical datasets or third party data, please ensure that the statement adheres to our policy

The mass spectrometry proteomics data have been deposited to the ProteomeXchange Consortium via the PRIDE partner repository with the dataset identifier PXD036857.

Human research participants

Policy information about studies involving human research participants and Sex and Gender in Research.

Reporting on sex and gender	*Use the terms sex (biological attribute) and gender (shaped by social and cultural circumstances) carefully in order to avoid confusing both terms. Indicate if findings apply to only one sex or gender; describe whether sex and gender were considered in study design whether sex and/or gender was determined based on self-reporting or assigned and methods used. Provide in the source data disaggregated sex and gender data where this information has been collected, and consent has been obtained for sharing of individual-level data; provide overall numbers in this Reporting Summary. Please state if this information has not been collected. Report sex- and gender-based analyses where performed, justify reasons for lack of sex- and gender-based analysis.*
Population characteristics	*Describe the covariate-relevant population characteristics of the human research participants (e.g. age, genotypic information, past and current diagnosis and treatment categories). If you filled out the behavioural & social sciences study design questions and have nothing to add here, write "See above."*
Recruitment	*Describe how participants were recruited. Outline any potential self-selection bias or other biases that may be present and how these are likely to impact results.*
Ethics oversight	*Identify the organization(s) that approved the study protocol.*

Note that full information on the approval of the study protocol must also be provided in the manuscript.

Field-specific reporting

Please select the one below that is the best fit for your research. If you are not sure, read the appropriate sections before making your selection.

☒ Life sciences ☐ Behavioural & social sciences ☐ Ecological, evolutionary & environmental sciences

For a reference copy of the document with all sections, see nature.com/documents/nr-reporting-summary-flat.pdf

Life sciences study design

All studies must disclose on these points even when the disclosure is negative.

Sample size	No statistical method was used to predetermine sample size. The n-numbers were determined consistent with standard practices in Drosophila studies.
Data exclusions	No samples were excluded.
Replication	All experiment were performed with at least three biologically independent replicates. All qRT-PCR data presented was additionally obtained with three technical replicates for each sample.
Randomization	Animals were allocated to experimental groups according to their genotype and developmental stage.
Blinding	The investigators were not blinded to allocation during the experiments and analysis.

Reporting for specific materials, systems and methods

We require information from authors about some types of materials, experimental systems and methods used in many studies. Here, indicate whether each material, system or method listed is relevant to your study. If you are not sure if a list item applies to your research, read the appropriate section before selecting a response.

Materials & experimental systems

n/a	Involved in the study
☐	☒ Antibodies
☐	☒ Eukaryotic cell lines
☒	☐ Palaeontology and archaeology
☐	☒ Animals and other organisms
☒	☐ Clinical data
☒	☐ Dual use research of concern

Methods

n/a	Involved in the study
☒	☐ ChIP-seq
☒	☐ Flow cytometry
☒	☐ MRI-based neuroimaging

Antibodies

Antibodies used

Rabbit Anti-GABA Type A Receptor -Associated Protein (GABARAPs) Abcam ab109364
Rabbit Anti-Microtubule-associated protein 1 light chain 3 (LC3B) Novus Biologicals 100-2220
Mouse Anti-Sequestosome 1 (SQSTM1/p62) Abnova H00008878-M01
Rabbit Anti-ZNRF2 Novus NBP1-28715
Rabbit Anti-VPS18 Abclonal A16654
Rabbit anti-VPS18 Abclonal A17563
Mouse Anti-Rab7 DSHB
Rabbit Anti-phospho-Akt 1:200 Cell Signaling 4054
Rabbit Anti-Cathepsin L/MEP Abcom ab58991
Mouse Anti-beta-Actin Sigma AM4302
Goat Anti-Green Fluorescent Protein (GFP) Rockford 600–101-215
Mouse Anti-Ubiquitin-horseradish peroxidase (HRP) Santacruz sc-8017
Mouse Anti-HA Cell Signaling CST.2367S (6E2)
Mouse Anti-Myc NEB 2276S (9B11)
Mouse Anti-FLAG Sigma F1804
Mouse Anti-V5 Abcam (ab27671)
Donkey Anti-Mouse AlexaFluor-488 Invitrogen A-21202
Donkey Anti-Mouse AlexaFluor-555 Invitrogen A-31570
Donkey Anti-Mouse AlexaFluor-647 Invitrogen A-31571
Donkey Anti-Rabbit AlexaFluor-488 Invitrogen A-21206
Donkey Anti-Rabbit AlexaFluor-555 Invitrogen A-31572
Donkey Anti-Goat IgG H&L (HRP) preadsorbed Abcam ab97120
Donkey Anti-Rabbit IgG HRP-linked whole Ab Amersham ECL NA934
Sheep Anti-Mouse IgG HRP-linked whole Ab Amersham ECL NA931

Validation

All antibodies used in this work have been validated as described by supplies in publications.

Eukaryotic cell lines

Policy information about cell lines and Sex and Gender in Research

Cell line source(s)

HeLa cells were obtained from Cellbank Australia and were from the European Collection of Authenticated Cell Cultures Collection Catalogue No.: 93021013.

S2 cells (S2-DRSC #181) were obtained from DGRC.

Authentication

The cells were not authenticated.

Mycoplasma contamination

The HeLa cell lines used were tested for mycoplasma contamination by MycoAlert (Lonza LT07-318) according to the manufacturer's instructions.

Commonly misidentified lines
(See ICLAC register)

Name any commonly misidentified cell lines used in the study and provide a rationale for their use.

Animals and other research organisms

Policy information about studies involving animals; ARRIVE guidelines recommended for reporting animal research, and Sex and Gender in Research

Laboratory animals

The animals used were Drosophila melanogaster and assayed at larval, prepupal or adult stage. The stocks used in the study: Mex-GAL4 was a gift from R Burke, hsFLP; pmCherry-Atg8a; Act>CD2>GAL4, UAS-nlsGFP/TM6B was a gift from E. Baehrecke, and the pmCherry-Atg8a was previously described (Denton, D. et al. 2012 Cell Death Differ 19, 1299-1307). The knockdown line CG14435R-1 (270R1), UAS-dor RNAi (3093R-2) and UAS-lt RNAi (18028R-2) were obtained from NIG-FLY and CG14435{GD8324} (v17600) from VDRC. The NP1-GAL4 (112001) and UAS-Atg1GS10797 line were obtained from Kyoto DGGR. Transgenic UAS-CG14435-EGFP lines were generated by BestGene Inc (CA, USA). The following stocks were from the Bloomington Drosophila Stock Center (Bloomington, IN, USA):
w1118 (BL3605)

w1118 Mi{GFP[E.3xP3]=ET1}CG14435MB05816 (BL25464)
w1118; snaSco/SM6a, P{w[+mC]=hsILMiT}2.4 (BL24613)
w1118 Df(1)BSC297/Binsinscy (BL23681)
UAS-ZNRF1:HA (BL79151)
UAS-ZRNF2 (BL86229)
UAS-lt RNAi (BL34871)
UAS-Vps16A RNAi (BL38271)
UAS-eGFP:Atg5 (BL59848)
UAS-GFP:LAMP1 (BL42714)
GMR-GAL4 (BL1104)

Wild animals	The study did not involve wild animals
Reporting on sex	The sex of the adult flies used in the study is detailed in the figure legend. For larval studies where appropriate males were used alternatively both male and females were used.
Field-collected samples	The study did not involve samples collected from the field.
Ethics oversight	No ethical approval was required. The study involves transgenic invertebrate laboratory animals (Drosophila melanogaster) and under the Australian Office of Gene Technology Regulator required physical containment to prevent spreading of transgenic animals in the environment.

Note that full information on the approval of the study protocol must also be provided in the manuscript.
